# Algorithms for automated diagnosis of cardiovascular diseases based on ECG data: A comprehensive systematic review

**DOI:** 10.1016/j.heliyon.2023.e13601

**Published:** 2023-02-10

**Authors:** Hanna Vitaliyivna Denysyuk, Rui João Pinto, Pedro Miguel Silva, Rui Pedro Duarte, Francisco Alexandre Marinho, Luís Pimenta, António Jorge Gouveia, Norberto Jorge Gonçalves, Paulo Jorge Coelho, Eftim Zdravevski, Petre Lameski, Valderi Leithardt, Nuno M. Garcia, Ivan Miguel Pires

**Affiliations:** a Instituto de Telecomunicações, Universidade da Beira Interior, 6200-001 Covilhã, Portugal; b Escola de Ciências e Tecnologia, University of Trás-os-Montes e Alto Douro, Quinta de Prados, 5001-801 Vila Real, Portugal; c Polytechnic of Leiria, Leiria, Portugal; d Institute for Systems Engineering and Computers at Coimbra (INESC Coimbra), Coimbra, Portugal; e Faculty of Computer Science and Engineering, University Ss Cyril and Methodius, 1000 Skopje, Macedonia; f VALORIZA, Research Center for Endogenous Resources Valorization, Instituto Politécnico de Portalegre, 7300-555 Portalegre, Portugal; g COPELABS, Universidade Lusófona de Humanidades e Tecnologias, Lisboa, Portugal

**Keywords:** Cardiovascular diseases, Systematic review, ECG sensors, Diagnosis, WHO, World Health Organization, ECG, Electrocardiography, AI, Artificial Intelligence, DL, Deep Learning, ML, Machine Learning, NLP, Natural Language Processing, CNN, Concolutional Neural Networks, SHAP, SHapley Additive exPlanations, SVM, Support Vector Machine, LASSO, Least Absolute Shrinkage and Selection Operator, MLR, Multiple Linear Regression, POAF, Postoperative Atrial Fibrillation, MLP, Multiplayer Perceptron, LR, Linear Regression, LSTM, Long Short-Term Memory, kNN, k-nearest neighbors, BNN, Binarized Neural Network, RNN, Recurrent Neural Network, GRU, Gated Recurrent Unit, DNN, Deep Neural Networks, GMM, Gaussian Mixture Model, GAN, Generative Adversarial Networks, GNB, Gaussian Naive bayes, LDA, Linear Discriminant Analysis, RF, Random Forest

## Abstract

The prevalence of cardiovascular diseases is increasing around the world. However, the technology is evolving and can be monitored with low-cost sensors anywhere at any time. This subject is being researched, and different methods can automatically identify these diseases, helping patients and healthcare professionals with the treatments. This paper presents a systematic review of disease identification, classification, and recognition with ECG sensors. The review was focused on studies published between 2017 and 2022 in different scientific databases, including PubMed Central, Springer, Elsevier, Multidisciplinary Digital Publishing Institute (MDPI), IEEE Xplore, and Frontiers. It results in the quantitative and qualitative analysis of 103 scientific papers. The study demonstrated that different datasets are available online with data related to various diseases. Several ML/DP-based models were identified in the research, where Convolutional Neural Network and Support Vector Machine were the most applied algorithms. This review can allow us to identify the techniques that can be used in a system that promotes the patient's autonomy.

## 1 Introduction

Cardiovascular diseases are one of the leading causes of death worldwide, and the World Health Organization (WHO) estimated that 17.9 million persons died in 2019 of this kind of disease [1]. Cardiovascular diseases are related to health problems that affect the heart and blood vessels [2]. The risk of these diseases increases with smoking, high blood pressure, high cholesterol, unhealthy diet, lack of exercise, and obesity [3].

The use of technology may improve healthcare treatments and monitoring with the help of different sensors available in the devices used daily by the population [4,5]. These devices, including smartphones, laptops, or tablets, allow the persons to be in constant contact with medical doctors, or they may have access to different information online [[6], [7], [8], [9], [10]]. It exploits the concepts of patient autonomy and empowerment, where each one can have tools to benefit the health. It is now included in a concept related to 5P-Medicine, where developing different devices will help healthcare in the future [11,12].

Regarding cardiovascular diseases, one crucial data to acquire from the individuals is their feedback [[13], [14], [15], [16]]. However, due to the low cost of some Electrocardiography (ECG) sensors, they can be used as a complement for the better visualization of the healthcare professionals [17,18]. Also, Artificial Intelligence is a computer science field that allows the creation of solutions for the automatic diagnosis of different diseases based on the data acquired from the sensors [[19], [20], [21]]. It included identifying different abnormal patterns of the data obtained from the sensors, allowing the identification of various diseases [[22], [23], [24], [25]]. Currently, this type of solution is scarce and under development, but it is essential to give autonomy to the patients [13,26,27]. Mainly, different datasets are related to various diseases identified by each capture [[28], [29], [30]]. A standard and secure application using data worldwide are essential to promote a considerable advance in healthcare treatments [31,32]. However, the General Data Protection Regulation must be considered.

As the ECG data is responsible for measuring heart rates and rhythms [33], it may be health healthcare professionals to check different healthcare problems remotely or create personalized medication for each individual. The world is constantly evolving, and various healthcare problems appear during this pandemic. Thus, the early detection of different diseases with machine learning methods may reduce its consequences [34].

The purpose of this study consists of a comprehensive systematic review of the different studies available in various scientific databases, including PubMed Central, Springer, Elsevier, Multidisciplinary Digital Publishing Institute (MDPI), IEEE Explore, and Frontiers published between 2017 and 2022, related to methods for disease identification, classification, and recognition with ECG sensors. This review is essential to understand the different usages of the ECG data and the techniques previously used in the literature to create a new methodology for the identification of diseases remotely.

Other reviews are available in the literature but need to cover the parameters in the data and the importance of ECG in cardiovascular disease diagnostics. The authors of [35] give an overview of the application of AI and describe AI-based approaches and focus only on the AI part of the diagnostics process. In Ref. [36], it is also given an overview of AI-based techniques and emphasizes deep learning-based AI. The authors of [37] also provide an overview of the analysis of algorithms for heart sounds. Other reviews exist in the literature. The main contribution of this review compared with the other reviews in the literature is that this review analyzes the global scope of algorithms for automated diagnosis and treats the approaches from both data acquisition and availability aspects and the application of the proposed methods on the diagnosis process.

## 2 Methodology

### 2.1 Research questions

This systematic review was focused on the following research questions: (RQ1) Which types of sensors can be used to track different diseases? (RQ2) Which ML/DP methods are mainly used primarily to support the automatic analysis of ECG data? (RQ3) Which diseases are specifically studied with the datasets available online? (RQ4) What challenges are related to monitoring different diseases with sensors?

### 2.2 Inclusion criteria

The research on disease identification, classification, and recognition with automatic methods based on ECG data was based on the following inclusion criteria: (1) studies that used ML/DP methods for measuring different parameters related to ECG data; (2) studies that are related to cardiovascular diseases; (3) studies that used different sensors to acquire ECG data or present a public dataset; (4) studies that find the importance of the ECG data considering for the monitoring of different diseases; (5) studies that include a concrete presentation of the purpose of the study; (6) studies with a defined population/dataset; (7) studies that show results (8) studies that were published between 2017 and 2022; (9) studies written in English.

### 2.3 Search strategy

This systematic review consisted of the research of studies based on disease identification, classification, and recognition with ECG data. The search was performed with a Natural Language Processing (NLP)-based framework [38] in the following databases: PubMed Central, Springer, Elsevier, Multidisciplinary Digital Publishing Institute (MDPI), IEEE Xplore, and Frontiers. The keywords applied for this research were: “ECG disease identification”, and “ECG disease classification”, “ECG disease recognition”. Each study was filtered using the defined criteria presented in Section 2.2. The research was performed on November 18, 2022.

### 2.4 Extraction of study characteristics

There are specific parameters extracted from the studies. The information from the studies was classified and presented in Table 1 by the following terms: year of publication, location, type of publication, population/dataset, the purpose of the studies, sensor/equipment, ML/DP applied methods, approach, and diseases. Some clarifications related to the studies were discovered by contacting the respective authors of the analyzed studies.

**Table 1:** Study analysis.

Paper	Year	Location	Type of Publication	Population/Dataset	Purpose of Study	Sensors	ML/DP Methods	Approach	Diseases
Anand et al. [39]	2022	New Delhi (India)	Journal article	PTB-XL dataset [28] (21837 samples from 18885 patients)	The implementation of a number of deep neural networks on a publicly available dataset of PTB-XL of ECG signals for the detection of cardiac disorders.	12-Lead ECG	CNN, SHAP	Patient-independent	Cardiovascular diseases
Geweid et al. [40]	2022	Michigan (USA)	Journal article	2017 Physionet/CinC challenge dataset [41] (8258 recordings)	A method based on a Hybrid Approach of Dual Support Vector Machine is used for the detection of atrial fibrillation	1-Lead ECG	SVM	Patient-independent	Atrial Fibrillation
Guo et al. [42]	2022	Xian (China)	Journal article	423 subjects from the International Cooperation Center for Hypertrophic Cardiomyopathy of Xijing Hospital	This work aimed to develop a pragmatic prediction model based on the most common ECG features to screen for Hypertrophic cardiomyopathy (HCM).	12-Lead ECG	LASSO, MLR	Patient-independent	Hypertrophic Cardiomyopathy
He et al. [43]	2022	Chengdu (China)	Journal article	100 patients from Department of Cardiovascular, West China Hospital of Sichuan University	This study aimed to build statistical models and machine learning models based on P-wave parameters to predict Postoperative Atrial Fibrillation (POAF).	12-Lead ECG	SVM	Patient-independent	Postoperative Atrial Fibrillation
Hsu et al. [44]	2022	Hualien (Taiwan)	Journal article	A population of 2,206 military males were obtained from the cardiorespiratory health in eastern armed forces study (CHIEF Heart Study) [45,46]	This study proposed a machine learning method for electrocardiographic features to identify Left Atrial Enlargement in young adults.	12-Lead ECG	MLP, SVM, LR	Patient-independent	Left Atrial Enlargement
Zhao al [47].	2022	Nanjing (China)	Journal article	MIT-BIH arrhythmia database [48] (48 patients)	An improved deep residual convolutional neural network is proposed to classify arrhythmias automatically	2-Lead ECG	CNN	Patient-independent	Arrhythmia
Liu et al. [49]	2022	Harbin (China)	Journal article	MIT-BIH arrhythmia database [48] (48 patients)	A network layer design based on LSTM to obtain the autoencoder structure is optimized. This structure can cooperate with the ECG preprocessing process designed by the same team to obtain better arrhythmia classification effects.	MLII (modified limb lead II) Lead	Auto-encoders, CNN, LSTM	Patient-independent	Arrhythmia
Mazidi et al. [50]	2022	Qeshm (Iran)	Journal article	MIT–BIH arrhythmia [48] database (22 records)	This study focused on the tunable Q-factor wavelet transform algorithm and statistical methods to detect PVC.	2-Lead ECG	SVM, kNN	Patient-independent	Premature Ventricular Contraction
Sawano et al. [51]	2022	Tokyo (Japan)	Journal article	29,859 patients	The development of a deep learning–based artificial intelligence algorithm for the diagnosis of significant aortic regurgitation using electrocardiography.	12-Lead ECG	CNN	Patient-specific	Aortic regurgitation
Zhao et al. [52]	2022	Guangzhou (China)	Journal article	1863 patients from Third Affiliated Hospital of Sun Yat-sen University, China	The propose of this study was to build a DL model based on convolutional neural networklong short-term memory (CNN-LSTM) to detect left ventricular hypertrophy	12-Lead ECG	CNN LSTM	Patient-independent	Left Ventricular Hypertrophy
Zheng et al. [53]	2022	Orange (USA)	Journal article	18612 ECG records extracted from 545 patients from Ningbo First Hospital of Zhejiang University	This work proposed an artificial intelligence-enabled ECG analysis algorithm to estimate possible origins of idiopathic ventricular arrhythmia at a clinical-grade level accuracy.	12-Lead ECG	Gradient Boosting	Patient-independent	Idiopathic Ventricular Arrhythmia
Dey et al. [54]	2021	Chittagong (Bangladesh)	Journal article	517 records of 268 individuals from Physikalisch-Technische Bundesanstalt (PTB) database [55], available in Physionet [56]	The development of a temporal feature-based classification approach for myocardial infarction based on merging of CNN e bi-LSTM methods.	12-Lead ECG	CNN, bi-LSTM	Patient-specific	Myocardial Infarction
Che et al. [57]	2021	Dalian (China)	Journal article	3699 records from males and 3178 from females	An end-to-end deep learning framework based on convolutional neural networks is proposed for ECG signal processing and arrhythmia classification.	12-Lead ECG	CNN	Patient-specific	Arrhythmia
Chen et al. [22]	2021	Wuhan (China)	Journal article	Intensive Care Medicine Database (61,532 patients)	A deep learning-based diagnosis system is proposed for the early detection of heart failure particularly in elderly patients.	2-Lead ECG	CNN	Patient-specific	Heart Failure
Dai et al. [58]	2021	Hsinchu (Taiwan)	Journal article	PTB Diagnostic ECG [29] database (233 subjects)	A deep convolutional neural network to classify five CVDs using standard 12-Lead ECG signals is proposed.	12-Lead ECG	CNN	Patient-independent	Cardiovascular diseases
Grogan et al. [59]	2021	Minnesota (USA)	Journal article	2541 patients	The development of an artificial intelligence-based tool to detect cardiac amyloidosis from a standard 12-lead electrocardiogram.	12-Lead ECG	kNN	Patient-specific	Amyloidosis
Haleem et al. [60]	2021	Coventry (UK)	Journal article	PhysioNet's QT [61] database, MIT-BIH Normal Sinus Rhythm [62] Database, BIDMC Congestive Heart Failure [63] Database, MIT-BIH Sudden Cardiac Death Holter [64] database, and MIT–BIH arrhythmia [48]	A two-stage multiclass algorithm is proposed. The first stage performs ECG segmentation based on Convolutional Bidirectional LSTM neural networks with attention mechanisms. A second stage is based on a time adaptive CNN applied to ECG beats extracted from the first stage for several time intervals.	2-Lead ECG	CNN, LSTM	Patient-independent	Cardiovascular diseases
Houssein et al. [65]	2021	Minia (Egypt)	Journal article	MIT–BIH arrhythmia [48] database (48 ECG records)	Different ECG signal descriptors based on one-dimensional local binary pattern, wavelet, higher-order statistical, and morphological information are introduced for feature extraction.	ECG sensors	SVM	Patient-independent	Arrhythmia
Hua et al. [66]	2021	Wuhan (China)	Journal article	40 thousand ECG samples from Hefei Hi-tech competition dataset [67]	The development of general feature extraction framework for ECG data, that can perform various kinds of feature engineering tasks, all of them have their meaning under a clinical context.	8-Lead ECG	BNN	Patient-independent	Arrhythmia
Jahmunah et al. [68]	2021	Jurong West (Singapore)	Journal article	Fantasia [69] and St. Petersburg [70] database	The development of an automated system for the automated categorization of ECG signals into normal, CAD, myocardial infarction and congestive heart failure classes using CNN and unique GaborCNN models.	2-Lead ECG	CNN	Patient-independent	Coronary Artery
Li et al. [71]	2021	Manchester (United Kingdom)	Journal article	6,877 (females: 3,178; males: 3,699) records collected from 11 hospitals	This study aimed to develop an auto-detection algorithm, which extracts valid features from 12-lead ECG for classifying multiple types of cardiac states.	12-Lead ECG	CNN, LSTM, BiLSTM, RNN	Patient-independent	Arrhythmia
Luo et al. [72]	2021	Kunming (China)	Journal article	MIT-BIH Atrial Fibrillation [73] database	The development of a mixed depth model for processing time series to predict multi-classification electrocardiographs.	2-Lead ECG	CNN, RNNs	Patient-independent	Arrhythmia
Naz et al. [74]	2021	Wah (Pakistan)	Journal article	MIT–BIH arrhythmia [48] database, CUDB arrhythmia [75] database, and Nsr arrhythmia database.	A deep learning approach is proposed for the detection of VA. Initially, the ECG signals are transformed into images that have not been done before. Later, these images are normalized and utilized to train the AlexNet, VGG-16 and Inception-v3 deep learning models.	2-Lead ECG	SVM	Patient-independent	Ventricular Arrhythmia
Nguyen et al. [76]	2021	Wellington (New Zealand)	Journal article	PhysioNet 2017 [41] dataset (8528 instances)	A method is proposed for recognition of AF from ECG signals by stacking a support vector machine on statistical features of segment-based recognition units produced by a convolutional neural network	1-Lead ECG	CNN, SVM	Patient-independent	Atrial Fibrillation
Radhakrishnan et al. [77]	2021	Hyderabad (India)	Journal article	Physionet Computing in Cardiology Challenge 2017 [41] dataset (8482 ECG records), MIT-BIH Atrial Fibrillation [73] database (25 ECG records) MIT–BIH arrhythmia [48] dataset (47 ECG records)	A time-frequency domain deep learning-based approach is proposed to detect AF and classify terminating and non-terminating AF episodes using ECG signals.	1-Lead ECG	CNN, DL, LSTM	Patient-independent	Atrial Fibrillation
Sabut et al. [78]	2021	Bhubaneswar (India)	Journal article	CUDB [75] and VFDB [79] databases	In this study, a VF/VT classification scheme is proposed using a deep neural network approach using hybrid time–frequency-based features.	ECG sensors	DL, ML	Patient-independent	Ventricular Arrhythmia
Yadav et al. [80]	2021	Raigad (India)	Conference Proceedings	The ECG signals for 200 individuals from PTB database [81]	This study developed an approach to diagnose MI using 7 -layer deep CNN automatically.	12-Lead ECG, 3-Lead Frank	CNN	Patient-specific	Myocardial Infarction
Wang et al. [82]	2021	Tianjin (China)	Journal article	MIT-BIH arrhythmia [48] database and China Physiological Signal Challenge (CPSC) 2018 [83]	This paper proposes an improved gated recurrent unit by setting a scale parameter into the existing bidirectional GRU model for PVC signals recognition.	ECG sensors	CNN, GRU	Patient-independent	Premature Ventricular Contraction
Wang et al. [84]	2021	Zhengzhou (China)	Journal article	CCDD [85] database	In order to better assist doctors in diagnosing cardiovascular diseases, a set of end-to-end automatic diagnosis algorithms for ECG diseases based on intelligent simulation modeling are proposed.	2-Lead ECG	DL, ML	Patient-independent	Cardiovascular diseases
Wu et al. [86]	2021	Wuhan (China)	Journal article	MIT–BIH arrhythmia [48] (48 ECG records)	A 12-layer deep one-dimensional convolutional neural network is proposed for classification of the five micro-classes of heartbeat types	2-Lead ECG	CNN, DL, Ensemble classifiers, ML	Patient-independent	Arrhythmia
Xiong et al. [87]	2021	Baoding (China)	Journal article	Physikalisch-Technische-Bundesanstalt (PTB) [29] database (290 subjects)	The development of a multi-lead MI localization approach based on the densely connected convolutional network.	12-Lead ECG	CNN	Patient-independent	Myocardial infarction
Zhang et al. [88]	2021	Beijing (China)	Journal article	CPSC 2018 dataset [83] (3,178 females and 3,699 males)	A deep learning classification method, namely, a global hybrid multi-scale convolutional neural network is proposed, to implement binary classification for AF detection.	1-Lead ECG	CNN, DL	Patient-independent	Atrial fibrillation
Zhang et al. [89]	2021	Jinjiang (China)	Journal article	MIT–BIH arrhythmia [48] database (48 ECG records)	This paper proposed a high-accuracy ECG arrhythmia classification method based on convolutional neural networks.	ECG sensors	CNN	Patient-independent	Arrhythmia
Zhang et al. [90]	2021	Ohio (USA)	Journal article	CPSC 2018 [83] dataset	The development a deep neural network for automatic classification of cardiac arrhythmias from 12-Lead ECG recordings	12-Lead ECG	DL, ML, SHAP	Patient-independent	Arrhythmia
Ambhore et al. [91]	2020	Texas (USA)	Journal article	MIT-BIH arrhythmia [48] database, BIDMC Congestive Heart Failure [63] database, MIT-BIH Normal Sinus Rhythm [62] Database	CVD detection by using Deep Neural Network with the help of Heart Rate Variability is proposed.	ECG sensors	CNN, DL, Ensemble classifiers, ML, SVM	Patient-independent	Cardiovascular diseases
Banerjee et al. [92]	2020	Vellore (India)	Conference Proceedings	8528 samples signals from Physionet Challenge 2017 CinC dataset [41]	This article aimed at developing a complete wearable application that use signal pre-processing combined with a Deep Learning Model consisting of 1-D Convolution Neural Networks and Long Short-term Memory Networks to classify single-lead ECG signals into different categories for early diagnosis of arrhythmia.	Single-Lead ECG	CNN, LSTM	Patient-specific	Arrhythmia
Banerjee et al. [93]	2020	Vellore (India)	Conference Proceedings	200 individuals were selected from MIMIC II waveform dataset [94] and the second dataset was created using real records from 150 individuals at the clinic environment	The authors proposed a neural network architecture based on hybrid CNN-LSTM model, that effectively combines two non-specific coronary artery disease markers, 1) anomalous morphology of Electrocardiogram (ECG) waveform and 2) abnormal Heart Rate Variability (HRV).	2-Lead ECG, Single-Lead ECG	CNN, LSTM	Patient-specific	Coronary Artery Disease
Bitarafan et al. [95]	2020	Tehran (Iran)	Conference Proceedings	Creighton university ventricular tachyarrhythmia database [75], MIT-BIH atrial fibrillation [96], and MIT-BIH arrhythmia databases [97]	This research work proposed a deep learning method based on hybrid DCNN-LSTM model for arrhythmia detection and classification that does not demand any heuristic segmentation.	ECG sensors	CNN, LSTM	Patient-specific	Arrhythmia
Bouny et al. [98]	2020	Mohammedia (Morocco)	Journal article	MIT-BIH Arrhythmia [48] database (randomly selected 45000 ECG fragments)	This paper presents an End-to-End Deep Learning method for heart disease diagnosis from single channel ECG signal.	ECG sensors	CNN, DL	Patient-independent	Cardiovascular diseases
Chumrit et al. [99]	2020	Chiang Rai (Thailand)	Conference Proceedings	MIT-BIH arrythmia, MIT-BIH normal sinus rhythm and QT database [56]	This study presented a method for arrhythmia detection from the ECG signal based on the average energy and zero-crossing quantities that are extracted from the ECG records.	ECG sensors	SVM	Patient-specific	Arrhythmia
Deng et al. [100]	2020	SuZhou (China)	Conference Proceedings	MIT-BIH atrial fibrillation database [56]	The proposed method based on time domain features of ECG sequence and one-dimensional CNN to detect atrial fibrillation.	ECG sensors	CNN, SVM	Patient-specific	Atrial Fibrillation
Hammad et al. [101]	2020	Minya (Egypt)	Conference Proceedings	48 ECG records obtained from 47 subjects composed of 47% female and 53% male participants from MIT-BIH database [56]	The proposed technique fused the adaptability and flexibility in input-output relationships of deep neural networks (DNN) models with the “learnability” of classical ML methods as well as repeatability inherent to the mutation, crossover and other properties of GA and other optimization techniques to realize efficient strategy for arrhythmia detection.	5-Lead ECG	DNN, kNN, SVM, MLP	Patient-specific	Arrhythmia
Hatamian et al. [102]	2020	Erlangen (Germany)	Conference Proceedings	8528 ECG signals from PhysioNet/CinC challenge 2017 [41] dataset	Was investigated the effectiveness of two most augmentation algorithms, namely Gaussian Mixture Model (GMM) and Generative Adversarial Networks (GANs) to identify the most suitable ones to enhance classification performance of Atrial Fibrillation in short ECG Signals.	Single-Lead ECG	GANs, GMM	Patient-specific	Atrial Fibrillation
Hsu et al. [103]	2020	San Diego (USA)	Conference Proceedings	MIT–BIH arrhythmia [48] database (48 ECG records)	A waveform-based signal processing (WBSP) technique was presented.	2-Lead ECG	DL, ML	Patient-independent	Arrhythmia
Jiang et al. [104]	2020	Guangzhou (China)	Journal article	12,000 adults those aged over 65 years old or diagnosed with atrial fibrillation (N = 3,585)	This study aimed to develop an artificial intelligence approach based on Convolutional Neural Network for the detection of Left atrial enlargement.	12-Lead ECG	CNN	Patient-independent	Left Atrial Enlargement
Ibrahim et al. [105]	2020	Abu Dhabi (United Arab Emirates)	Journal article	713,447 extracted ECG samples and associated auxiliary data ECG-ViEW II [106] database	This study proposed framework to predict the onset of Acute Myocardial Infarction realized with two deep learning models, a convolutional neural network (CNN) and a recurrent neural network (RNN), and a decision-tree based model, XGBoost.	12-lead ECG	RNN, CNN, XGBoost	Patient-specific	Acute Myocardial Infarction
Li et al. [107]	2020	Beijing (China)	Journal article	MIT–BIH arrhythmia [48] database (48 ECG records)	This paper proposed designing a simple architecture of deep neural network, CraftNet, for accurately recognizing the handcraft features.	2-Lead ECG	DL, Ensemble classifiers	Patient-independent	Cardiovascular diseases
Li et al. [108]	2020	Zhuhai (China)	Conference Proceedings	48 ECG signals from PhysioBank MIT-BIH arrhythmia [88] database	An ECG classification model by using a CNN-based broad learning system (CNNBLS) for automatic recognition of arrhythmia was proposed.	MLII and V5-Lead ECG	CNN, LSTM,	Patient-specific	Arrhythmia
Liang et al. [109]	2020	Guilin (China)	Journal article	MIT–BIH arrhythmia [48] Database (1000 10-s single-lead ECG segments), ICBEB [24] dataset (6,877 records) and PhysioNet Challenge 2020 [110] database	A deep learning algorithm for exploring the heartbeat event classification was proposed, and a systemic comparison based on the different methods and databases was conducted.	1-Lead and 12-Lead ECG	CNN, DL, LSTM	Patient-independent	Cardiovascular diseases
Prabhakararao et al. [111]	2020	Guwahati (India)	Journal article	data consisted of 124 MI patients, 49 HC and 41 (26 MMI patients and 15 with other cardiac diseases) non-MI patients from PTB diagnostic database [56]	A method based on neural network with intra- and inter-lead attention modules for automated diagnosis of MI form non-MI patients using 12-lead ECG and patients' clinical features was presented.	12-Lead ECG	RNN	Patient-independent	Myocardial Infarction
Mazaheri et al. [112]	2020	Isfahan (Iran)	Journal article	MIT–BIH arrhythmia [48] database (45 patients)	A computer-aided diagnosis system was provided for the automated classification and diagnosis of seven types of cardiac arrhythmias using the ECG signal.	2-Lead ECG	ML, kNN	Patient-independent	Arrhythmia
Rahman et al. [113]	2020	Nashville (USA)	Conference Proceedings	48 records of two channels ECG signals collected from 47 individuals obtained from MIT-BIH Arrhythmia [48] database	DP 1-D CNN classification model was proposed for the automatic ECG heartbeat for five distinct types of cardiac arrhythmia.	ECG sensors	CNN	Patient-specific	Arrhythmia
Subramanian et al. [114]	2020	Coimbatore (India)	Conference Proceedings	MIT-BIH database [48]	This work proposed an SVM based solution that classifies ECG data into types of arrhythmias.	ECG sensors	SVM	Patient-specific	Arrhythmia
Wang et al. [115]	2020	Shanghai (China)	Journal article	MIT-BIH arrhythmia [48] database (25 men and 22 women)	A dual fully connected neural network model for accurate classification of heartbeats was presented.	2-Lead ECG	CNN	Patient-independent	Arrhythmia
Wang et al. [116]	2020	Zhengzhou (China)	Journal article	PTB MI ECG [29] database (290 subjects)	The aim of the paper was to provide a method to detect MI leveraging ECG.	12-Lead ECG	Ensemble classifiers, SVM, kNN	Patient-independent	Myocardial Infarction
Yang et al. [117]	2020	Changchun (China)	Journal article	MIT-BIH Normal Sinus Rhythm [62] Database, St Petersburg INCART 12-lead Arrhythmia [70] Database, and BIDMC Congestive Heart Failure [63] Database.	A CAD and CHF classification method based on ECG fragment alignment was proposed -principal component analysis convolutional network.	12-Lead ECG	CNN, SVM	Patient-independent	Coronary Artery
Yao et al. [118]	2020	Jinan (China)	Journal article	Included dataset of 107 healthy control and 93 CAD patients, collected by Shandong Provincial Qianfoshan Hospital between November 2017 and September 2019	Aimed to explore the efficacy of the information derived from QT interval time-series and ST–T segment waveforms in ECG based automated CAD detection, an automated diagnostic system for CAD was developed.	Single-Lead ECG	GNB, RNN	Patient-specific	Coronary Artery
Zhang et al. [119]	2020	Hefei (China)	Journal article	ICBEB [24] dataset (6,877 subjects)	A spatio-temporal attention-based convolutional recurrent neural network was proposed to focus on representative features along both spatial and temporal axes.	12-Lead ECG	CNN, DL, RNNs	Patient-independent	Arrhythmia
Bashar et al. [120]	2019	Berlin (Germany)	Conference Proceedings	36 subjects from Medical Information Mart for Intensive [94] database	This study presented a method to detect AF from ICU patients using the MIMIC III ECG waveform.	ECG sensors	SVM, LDA, kNN	Patient-specific	Atrial fibrillation
Boppana et al. [121]	2019	Chennai (India)	Conference Proceedings	PhysioNet Database [81] and Apnea ECG image Database	This study proposed a system to conquer the disadvantage of standard system, by translating the exact ECG by utilizing machine perception and to identify the problems in ECG, mainly concentrates on OSA as well as Myocardial infraction.	ECG sensors	K-Medoids, kNN	Patient-independent	Obstructive sleep Apnea (OSA), Myocardial infraction
Celin et al. [122]	2019	Coimbatore (India)	Conference Proceedings	MIT-BIH Arrhythmia [48] database	The presented paper presented an automated long-term ECG signal analysis and classification methodology based on RF classifier.	2 -Leads ECG	SVM, Adaboost, ANN, RF	Patient-specific	Arrhythmia
Deb et al. [123]	2019	Khulna (Bangladesh)	Conference Proceedings	36 signals from MIT-BIH Normal Sinus Rhythm Database [62] and 36 signals from PTB Diagnostic [29] ECG Database	The purpose of this work aimed to classify ECG signals into normal and abnormal cardiac conditions groups using SVM algorithm.	12-Lead ECG, 3 Frank lead ECG	SVM	Patient-independent	Abnormal cardiac conditions
Gao et al. [124]	2019	Zhengzhou (China)	Journal article	MIT-BIH arrhythmia [48] database (48 patients)	A LSTM with FL was proposed to handle imbalanced ECG beat data on the MIT-BIH arrhythmia database.	ECG sensors	LSTM	Patient-independent	Arrhythmia
Hoang et al. [125]	2019	Hsinchu (Taiwan)	Conference Proceedings	32 subjects (75 records) from Physionet St. Petersburg Institute of Cardiological Technics [110]	A multi-leads ECG premature ventricular contraction detection method using tensor decomposition and Convolutional Neural Network was proposed.	6-Lead ECG, 12-Lead ECG	CNN	Patient-specific	Premature Ventricular Contraction
Kong et al. [126]	2019	Shanghai (China)	Journal article	1056 AF patients and 904 healthy people	In this study, an improved machine learning method was proposed for rapid modeling and accurate diagnosis of AF.	Inno-12-U ECG	ML, SVM	Patient-specific	Atrial Fibrillation
Mahmood et al. [127]	2019	Khartoum (Sudan)	Conference Proceedings	Physionet Computing in Cardiology Challenge 2017 dataset [128]	Comparison study with the purpose to propose an approach for selecting the best classifier for diagnoses of atrial fibrillation (AF) in coronary heartbeats.	1-Lead (LA-RA) ECG	kNN, SVM, Decision trees, Random Forest, AdaBoost ensemble classifier	Patient-specific	Atrial Fibrillation
Li et al. [129]	2019	Taiyuan (China)	Journal article	Chest pain centers (CPCs) of Shanxi Academy of Medical Sciences dataset (573 patients)	A deep convolutional neural network-Recurrent neural network model for automatic staging of heart failure diseases in real-time and dynamically was proposed.	2-Lead ECG	CNN, DL, ML, RNNs	Patient-specific	Heart Failure
Nankani et al. [130]	2019	Assam (India)	Conference Proceedings	8528 ECG records from PhysioNet Computing in Cardiology Challenge 2017 database [128]	The propose of this study was the development of end-to-end framework for automatic detection of Atrial Fibrillation using Deep Residual Learning.	Single-Lead ECG	CNN, Wider CNN, ResNet, CRNN	Patient-specific	Atrial Fibrillation
Pandey et al. [131]	2019	Raipur (India)	Journal article	MIT–BIH arrhythmia [48] database	An 11-layer deep convolutional neural network model was proposed for classification of the MIT-BIH arrhythmia database into five classes according to the ANSI–AAMI standards.	ECG sensors	CNN	Patient-independent	Arrhythmia
Prabhakararao et al. [132]	2019	Assam (India)	Conference Proceedings	549 records from 290 subjects with varying cardiac abnormalities from PhysioNet/PTBDB diagnostic database [56]	This study proposed a weighted SVM-based approach for automatic detection of posterior myocardial infarction using VCG signals.	12-lead ECG, 13-Lead VCG	SVM	Patient-specific	Posterior Myocardial Infarction
Tadesse et al. [133]	2019	Berlin (Germany)	Conference Proceedings	ICBEB [24] dataset (6,877 subjects)	An end-to-end trainable cross-domain transfer learning was proposed for CVD classification from ECG waveforms, by utilizing existing vision-based CNN frameworks as feature extractors, followed by ECG feature learning layers.	12-Lead ECG	CNN, DL	Patient-independent	Cardiovascular diseases
Tison et al. [134]	2019	Boston (USA)	Journal article	University of California, San Francisco [135] database (36186 ECGs records)	The development and testing of an algorithmic framework that facilitates scalable analysis of ECG data while preserving interpretable parallels to cardiac physiology.	12-Lead ECG	CNN, ML	Patient-independent	Cardiovascular diseases
Tripathy et al. [136]	2019	Hyderabad (India)	Journal article	Medical Center (BIDMC) CHF [63] database and MIT-BIH arrhythmia [48] database.	This paper proposed an approach to design a classifier-based system for the automated detection of CHF.	2-Lead ECG	kNN	Patient-independent	Congestive Heart Failure
Wang et al. [137]	2019	Shanghai (China)	Journal article	MIT–BIH arrhythmia [48] database (48 ECG records)	An improved CNN was proposed to automatically classify the heartbeat of arrhythmia.	2-Lead ECG	CNN, ML	Patient-independent	Arrhythmia
Wang et al. [138]	2019	Shenzhen (China)	Conference Proceedings	ICBEB [24] dataset (6,877 subjects)	An end-to-end deep learning method for multiclass arrhythmia detection with multiple stage features fusion was proposed.	12-Lead ECG	CNN	Patient-specific	Arrhythmia
Wu et al. [139]	2019	Shandong (China)	Journal article	Cleveland Heat Disease [140] database and Statlog Heart Disease [140] database	A method that uses K-Nearest Neighbor to impute missing values of ECG data and Z-score to standardize ECG data for the requirement of the random forest was proposed. This study combined the random forest and ECG data to develop an ECG left ventricular hypertrophy classifier.	ECG sensors	Ensemble classifiers, kNN	Patient-independent	Left Ventricular Hypertrophy
Zhang et al. [141]	2019	Nanjing (China)	Journal article	ECG management system of the First Affiliated Hospital of Nanjing Medical University (277,807 ECGs records)	A deep learning method was applied to build a system for automated detection and classification of ECG signals.	12-Lead ECG	CNN, DL	Patient-specific	Cardiovascular diseases
Abdeldayem et al. [142]	2018	Morgantown (USA)	Conference Proceedings	48 patients' records from MIT-BIH arrhythmia database [48]	The propose of this study was a development of a machine learning approach that augments the traditional arrhythmia detection approaches via our automatic arrhythmia classification system.	2- and 6- Leads ECG	SVM, kNN	Patient-independent	Arrhythmia
Ebrahimzadeh et al. [143]	2018	Tehran (Iran)	Journal article	Atrial Fibrillation Prediction [144] Database (106 records)	A method was proposed for the prediction of the onset of PAF, through integrating classical and modern methods	ECG sensors	ML	Patient-independent	Atrial Fibrillation
Gomes et al. [145]	2018	São João del-Rei (Brazil)	Journal article	Guvenir et al. dataset [146]	This paper combined several data mining techniques, such as clustering, feature selection, oversampling strategies, and automatic classification algorithms to create more efficient classification models to identify the disease.	ECG sensors	Ensemble classifiers, ML	Patient-independent	Arrhythmia
Hammad et al. [23]	2018	Harbin (China)	Journal article	MIT–BIH arrhythmia [48] dataset (25 men and 22 women)	A classifier that simulates the diagnosis of the cardiologist to classify the ECG signals into normal and abnormal from a single lead ECG signal was proposed.	2-Lead(MLII) ECG and lead V1	SVM, kNN	Patient-independent	Abnormal Heart Conditions
Hao et al. [147]	2018	Honolulu (Hawaii)	Conference Proceedings	MIT–BIH atrial fibrillation [73] database	A classification method called Softmax regression model was proposed, and it uses the known state data of two-layer neural network structure of the Softmax regression model for training and learning, and then calculates the probability of reclassification data belonging to each category.	ECG sensors	kNN	Patient-independent	Cardiovascular diseases
Iqbal et al. [148]	2018	Kuala Lumpur (Malaysia)	Journal article	MIT-BIH dataset, and University of Malaya Medical Center dataset	An approach called deep deterministic learning was proposed, which works by combining predefined heart activities with fused datasets to classify MI and Af.	2-Lead ECG	DL	Patient-independent	Myocardial infarction Atrial fibrillation
Liu et al. [149]	2018	Ansan (Korea)	Conference Proceedings	UCR Time Series Archive [150] dataset	A classification method of heart diseases based on ECG by adopting a machine learning method, called Long Short-Term Memory (LSTM) was proposed.	ECG sensors	DL, LSTM, ML	Patient-independent	Cardiovascular diseases
Mukherjee et al. [25]	2018	Kolkata (India)	Journal article	PhysioNet 2017 Challenge public database [41] (8528 ECG recordings)	Developing an algorithm for classification of short, single lead Electrocardiogram recordings into normal, AF, other abnormal rhythms and noisy classes	AliveCor device (1-Lead ECG)	Ensemble classifiers	Patient-independent	Atrial fibrillation
Raj et al. [151]	2018	Bihta (India)	Journal article	MIT–BIH arrhythmia [48] database	This study presented a technique for representation of electrocardiogram signals using sparse decomposition using a composite dictionary.	ECG sensors	SVM	Patient-independent	Arrhythmia
Raj et al. [152]	2018	Bihta (India)	Journal article	MIT–BIH arrhythmia [48] database (48 ECG records)	The proposal of a feature extraction method using the sparse representation technique to represent the different ECG signals for analysis.	2-Lead ECG	SVM, kNN	Patient-independent	Arrhythmia
Warrick et al. [153]	2018	Montreal (Canada)	Journal article	PhysioNet/CinC Challenge 2017 [41] database (8528 ECG recordings)	This work aimed to construct an intelligent tool that assists cardiologists in identifying cardiac arrhythmias and noise in electrocardiogram recordings.	1-Lead ECG	CNN, Ensemble classifiers, LSTM	Patient-independent	Arrhythmia
Wu et al. [154]	2018	Taipei (Taiwan)	Conference Proceedings	202 ECG records from MIT-BIH-AR database and 897 recordings from DeepQ database [155]	This study presented an end-to-end generic ECG heartbeat classification model that addresses interpatient variability and achieves the state-of-the-art performance for arrhythmia detection on the MIT-BIH-AR database.	2-Lead ECG, single-Lead ECG	CNN	Patient-specific	Arrhythmia
Xu et al. [156]	2018	Jinan (China)	Journal article	MIT-BIH Atrial Fibrilation [73] database (25 recordings)	A deep CNN with a total of 12 layers was developed to train an AF/non-AF classification model.	ECG sensors	CNN	Patient-independent	Atrial Fibrillation
Zhang et al. [157]	2018	Jinan (China)	Conference Proceedings	48 ECG records from 47 subjects from MIT-BIH database [48]	In this paper, was propose a nine-layer convolutional neural network (CNN) that can automatically extract appropriate features and detect the different categories of ECG beats based on individual records.	1-Lead ECG	CNN	Patient-independent	Arrhythmia
Acharya et al. [158]	2017	Singapore	Journal article	St. Petersburg Institute of Cardiological Technics 12-lead Arrhythmia [70] Database and Fantasia open access [69] database	This work proposed application of Higher-Order Statistics and Spectra for an automated classification of normal and CAD conditions using ECG signals.	12-Lead ECG	kNN	Patient-independent	Coronary artery
Andreotti et al. [159]	2017	Oxford (United Kingdom)	Conference Proceedings	8,528 ECG segments from Physionet/Computing in Cardiology Challenge 2017 database [128]	This study presented a comparison of a feature-based and a deep learning approach to classify rhythms from short ECG segments.	Single-Lead ECG	CNN, RNNs	Patient-specific	Atrial Fibrillation
Couceiro et al. [160]	2017	Coimbra (Portugal)	Conference Proceedings	12 patients	Model based on SVM algorithm for AF detection was proposed.	12-Lead ECG	SVM	Patient-specific	Atrial Fibrillation
Dolatabadi et al. [161]	2017	Tehran (Iran)	Journal article	46 men and 29 women	A method for the automatic diagnosis of normal and coronary artery disease conditions using Heart Rate Variability signals extracted from electrocardiogram was proposed.	ECG sensors	SVM	Patient-specific	Coronary Artery
Khatun et al. [162]	2017	Memphis (USA)	Conference Proceedings	440 records from PTB Diagnostic ECG database [81]	This work focused on the detection of MI and AR from single-lead ECG data applying ML technique and compares different lead performances in order to find the most suitable lead so that any of these two diseases can be detected using a single classifier with minimum delay.	Single-Lead ECG	Bagging Tree	Patient-specific	Myocardial Infarction, Arrhythmia
Pławiak et al. [163]	2017	Krakow (Poland)	Journal article	MIT–BIH arrhythmia [48] database (45 patients)	This article presented a research methodology that enables the classification of cardiac disorders based on ECG signal analysis and an evolutionary-neural system.	1-Lead ECG	SVM	Patient-independent	Cardiovascular diseases
Pławiak et al. [164]	2017	Coimbatore (India)	Journal article	MIT–BIH arrhythmia [48] database (29 patients)	This article presented a genetic ensemble of classifiers applied to classification of cardiac disorders based on ECG signal analysis.	1-Lead ECG	Ensemble classifiers	Patient-independent	Myocardial infarction
Plesinger et al. [165]	2017	Brno (Czech Republic)	Conference Proceedings	Physionet Challenge 2017 database [128]	This study presented a method for automated classification of holter ECG recordings into four groups: normal recordings, recordings with atrial fibrillation, recordings with any other arrhythmia, and noisy recordings.	1-Lead ECG	CNN	Patient-specific	Atrial Fibrillation, Arrhythmia
Shimpi et al. [166]	2017	Mumbai (India)	Conference Proceedings	279 different attributes from the cardiac arrhythmia dataset of the UCI machine learning repository [167]	This paper introduced an approach to classify the ECG data into one of the sixteen types of arrhythmia using Machine Learning.	ECG sensors	Random Forest, SVM, Logistic Regression and kNN	Patient-specific	Arrhythmia
Soliński et al. [168]	2017	Warsaw (Poland)	Conference Proceedings	8528 ECG records from PhysioNet Challenge database [128]	This study aimed to develop machine learning based algorithm for classification of AF and other rhythms from short-term signal.	Single-Lead ECG	ANN	Patient-specific	Atrial Fibrillation
Tan et al. [169]	2017	Singapore	Journal article	Fantasia [69] and St Petersburg Institute of Cardiology Technics [70] database (7 CAD and 40 normal subjects)	The implementation of a long short-term memory network with a convolutional neural network to automatically diagnose CAD ECG signals was proposed.	2-Lead ECG	CNN, DL, LSTM	Patient-independent	Coronary Artery
Warrick et al. [170]	2017	Montreal (Canada)	Conference Proceedings	PhysioNet Challenge 2017 dataset [128] which consisted of 8528 ECG signals	A deep learning model, named CL3, for automatic classification of cardiac arrhythmias based on raw single lead ECGs was proposed	single-Lead ECG	CNNc, LSTM	Patient-specific	Arrhythmia

## 3 Results

**Fig. 1:**
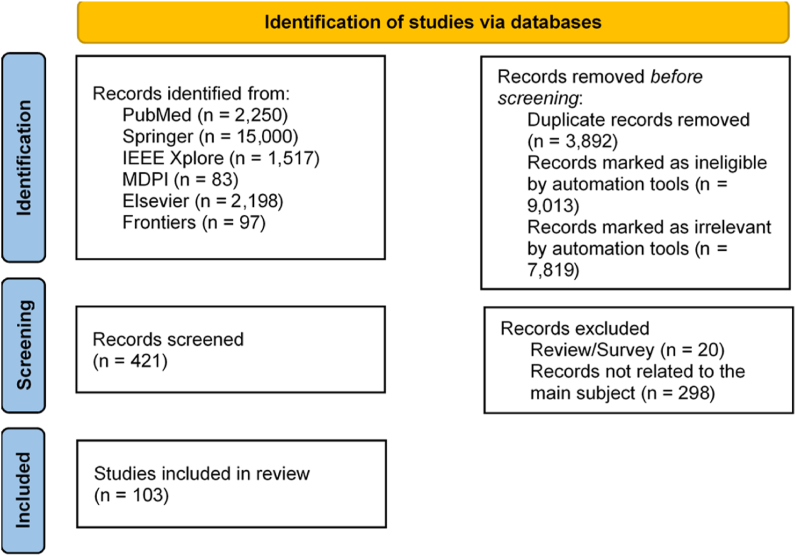
Flow diagram of identification and inclusion of papers.

As presented in Fig. 1, this systematic review found 21145 articles, of which 3892 are duplicates, 9013 are identified as incomplete sources, and 7819 are marked as irrelevant by automation tools. The remaining 421 studies were manually analyzed. In the analysis and full-text evaluation, we removed 20 papers that were Review/Survey, and 298 papers were not related to the main subject. The remaining 103 papers were included in the qualitative and quantitative synthesis. In summary, we examined 103 scientific articles. The reader must check the original published works for more information relevant to the different studies.

### 3.1 Quantitative analysis

Based on the results presented in Table 1, the analyzed studies were published between 2017 and 2022, reporting 11 studies in 2022 (10.7%), 23 studies in 2021 (22.3%), 24 studies in 2020 (23.3%), 19 studies in 2019 (18.4%), 14 studies in 2018 (13.6%), and 12 studies in 2017 (11.7%). Based on the locations of the different studies, 35 studies were conducted in China (34.0%), 19 studies were performed in India (18.4%), 10 studies were performed in the USA (10.1%), 5 studies were conducted in Iran (4.9%), 4 studies were performed in Taiwan (3.9%), with 3 studies each country (2.9%) were performed in Germany, Singapore, and UK, with 2 studies each country (1.9%) were performed in Bangladesh, Canada, Egypt, and Poland, and with 1 study each country (1.0%) was performed in Brazil, Czech Republic, Hawaii, Japan, Korea, Malaysia, Morocco, New Zealand, Pakistan, Portugal, Sudan, Thailand, and United Arab Emirates. Regarding types of publication sources, 34 studies (67.0%) are from Conference Proceedings, and the remaining 69 studies (33.0%) are from Journal papers. Some important sensors were used to perform the studies, 26 studies used only a 12-lead ECG sensor (25.2%) and 5 studies used a 12-lead ECG, complemented with other leads configurations (4.9%), 20 studies used a solo 2-Lead ECG sensor (19,4%) and 4 studies used a 12-lead ECG, complemented with other leads configurations (4.9%), 19 studies used 1-lead sensor (18.4%), 2 studies used MLII (Modified Limb Lead II) lead sensor (2.0%), 1 study used 8-lead ECG sensor (1.0%), 1 study used 5-lead ECG sensor (1.0%), 1 study used Inno-12-U ECG sensor (1.0%) and the remaining 24 studies did no lead sensors specification specified (23.3%). In addition, all the studies used machine learning methods. Many of these (64.1%) are applied to patient-independent methods, indicating that most studies explore models that offer accurate performance across multiple subjects. The remaining 35.9% are patient-specific methods designed considering personalized data from a specific patient. Regarding the different techniques implemented, 45 studies (43,7%) used a single method, where 18 studies (40,0%) referred to the use of CNN, 12 studies (2.7%) referred to the use of SVM, 4 studies (0.9%) referred to the use of kNN, 2 studies (0.4%) referred to the use of Ensemble classifiers, 1 studies (0.2%) each referred to the use of ANN, Bagging Tree, BNN, CNN, DL, Gradient Boosting, LSTM, and ML, and 38 studies (56,3%) used more than one method, where at least, 19 studies referred to the CNN method, 13 studies referred to the ML method, 12 studies referred to the SVM method, 10 studies referred to the DL method, 9 studies referred to each of the kNN, and LSTM methods, 7 studies referred to the Ensemble classifiers method, 3 studies referred to the RNN method, 2 studies referred one of the methods in closed in the following techniques, such as LR, SHAP, CNN, MLP, and Random Forest, and 1 study referred one of the methods in closed in the following methods, such as bi-LSTM, CNNc, GANs, GMM, GNB, GRU, K-Medoids, LASSO, MLR, Auto-encoders, LDA, Logistic Regression, kNN, XGBoost, Adaboost, ANN, BiLSTM, CRNN, DNN, ResNet, RF, RNN, Wider CNN, AdaBoost ensemble classifier, and Decision trees. The studies also considered different types of diseases, 36 studies were based on arrhythmia (35.0%), 17 studies were based on atrial fibrillation (16.5%), 14 studies on cardiovascular diseases (13.6%), 7 studies each were based on coronary artery and myocardial infarction (7.2%), 3 studies were based on premature ventricular contraction (2.9%), 2 studies each were based on abnormal cardiac conditions, heart failure, left atrial enlargement, left ventricular hypertrophy and ventricular arrhythmia (1.9%), and 1 study each was based on acute myocardial infarction, amyloidosis, aortic regurgitation, congestive heart failure, hypertrophic cardiomyopathy, idiopathic ventricular arrhythmia, posterior myocardial infarction, and postoperative atrial fibrillation.

To simplify the qualitative analysis, Table 2 presents the combination of studies by pathology similarity into 3 groups. The Change in heart rate group aggregates 57 studies (55.3%) related to changes in the rhythm of the heartbeat. The Myocardial dysfunction/pathologies group encloses 14 studies (13.6%) associated with the functioning of the myocardium muscle, which is responsible for pumping blood to the rest of the body. The last group is more generic and comprehensive, aggregating all the other 32 studies (31.1%) reported that do not fit in the previously presented groups.

**Table 2:** Grouping of the studies by the similarity of pathology.

Group nomenclature	Pathologies present in the studies
Changes in heart rate	Arrhythmia, Atrial fibrillation, Idiopathic ventricular arrhythmia, Postoperative atrial fibrillation, Ventricular arrhythmia
Myocardial dysfunction/pathologies	Acute myocardial infarction, Left ventricular hypertrophy, Myocardial infarction, Posterior myocardial infarction, Premature ventricular contraction
Other cardiovascular pathologies	Abnormal cardiac conditions, Amyloidosis, Aortic regurgitation, Cardiovascular diseases, Congestive heart failure, Coronary artery, Heart failure, Hypertrophic cardiomyopathy, Left atrial enlargement, Obstructive sleep apnea (OSA)

### 3.2 Changes in heart rate

Mukherjee et al. [25] present an algorithm for classifying short single-lead algorithms into four classes: Normal, Atrial Fibrillation, Other abnormal rhythms, and noisy. To test this method, the authors sourced from the PhysioNet 2017 Challenge public database a dataset of short single-lead ECG signals collected using an AliveCor device. This database comprises 8528 ECG recordings, of which 5050 would be classified as normal, 738 as Atrial Fibrillation, 2456 as other arrhythmias, and 284 as noisy data. From these signals, the authors extracted a total of 188 features. The method presented in this study earned the authors a top position in the PhysioNet 2017 Challenge, achieving, for the hidden test data, a 92% F1-score in the detection of AF recordings, an 86% F1-score in the detection of Normal rhythms, a 74% F1 score in the detection of other types of abnormal rhythms, and an overall F1-score of 83%.

In [40], the top five methods for Atrial Fibrillation detection submitted in the PhysioNet/Computing in Cardiology Challenge 2017 were compared, and a method based on a Hybrid Approach of Dual Support Vector Machine (HA-DSVM) for Atrial Fibrillation detection is proposed. The authors tested the method on the dataset collected from the 2017 Physionet/CinC Challenge, which contains 8258 recordings of single-lead ECG signals. Of these 8258 recordings, 5145 were normal sinus rhythms, 2557 were other rhythms, 71 were Atrial Fibrillation, and 46 were noisy recordings. From these recordings, the authors extracted the following features: R-wave, QRS-Waves, P-R Interval, Q-T Interval, S-T Interval, P, QRS, and T Waves, Normal rhythm, AFr rhythm, and Noisy rhythm. When applying this method, the authors achieved a precision of 70.80%, a specificity of 57.18%, an accuracy of 99.40%, and an F1-score of 85.30%.

He et al. [43] collected long-term ECG data 24 h before surgery and 7 days after surgery by single-lead ECG from 100 patients with preoperative sinus rhythm who underwent cardiac surgery. The patients were divided into a Postoperative atrial fibrillation (POAF) group and a no-POAF group. A clinical model and a clinical + ECG model were constructed. The ECG parameters were designed, and a support vector machine (SVM) was selected to build a machine-learning model and evaluate its prediction efficiency. The detection rate of POAF in long-term ECG monitoring was 31%, and that in conventional monitoring was 19%. We calculated 7 P-wave parameters, Pmax (167 ± 31 ms vs. 184 ± 37 ms, P = 0.018), Pstd (15 ± 7 vs. 19 ± 11, P = 0.031), and PWd (62 ± 28 ms vs. 80 ± 35 ms, P = 0.008) were significantly different. The AUC of the clinical model (sex, age, LA diameter, GFR, mechanical ventilation time) was 0.86. Clinical + ECG model (sex, age, LA diameter, GFR, mechanical ventilation time, Pmax, Pstd, PWd), AUC was 0.89. The machine learning model's accuracy (Ac) of the train set and test set was above 80 and 60%, respectively.

Hsu et al. [44] applied three machine learning classifiers, including the multilayer perceptron (MLP), logistic regression (LR), and support vector machine (SVM) with a linear kernel, were used for 26 ECG features and with or without six biological training to identify the presence of Left atrial enlargement (LAE) from 2,206 male adults aged 17–43 years in Taiwan. The definition of LAE was based on an echocardiographic left atrial dimension >4 cm in the parasternal long-axis window. The most significant area under the receiver operating characteristic curve is present in machine learning of the SVM for ECG only (77.87%) and of the MLP for all biological and ECG features (81.01%), both of which are superior to the P wave duration (62.19%). If the sensitivity is fixed to 70–75%, the specificity of the SVM for ECG only is up to 72.4%, and that of the MLP for all biological and ECG features is increased to 81.1%, both of which are higher than 48.8% by the P wave duration.

The authors of [47] proposed a deep residual convolutional neural network to classify arrhythmias automatically. The ECG signals used in the study were retrieved from the MIT-BIH arrhythmia database, with 48 recordings selected, each with a 30-min duration. This method does not require feature extraction in a specific domain but extracts the ECG features automatically. The method proposed by the authors achieved a sensitivity of 94.54%, a positive predictivity of 93.33%, and a specificity of 80.80% for normal segments; a sensitivity of 35.22%, a positive predictivity of 65.88%, and a specificity of 98.3% for the supraventricular ectopic segment; and a sensitivity of 88.35%, a positive predictivity of 79.86%, and a specificity of 94.92% for the ventricular ectopic segments.

The authors of [49] optimized a network layer design based on Long Short-Term memory to obtain the autoencoder structure. In the same amount, the ECG signal data utilized during the study was extracted from the MIT-BIH arrhythmia database and the MIT-BIH supraventricular arrhythmia database. The ECG features from these signals are extracted automatically by the autoencoder. The method proposed by the authors achieved an accuracy of 98.50%, a sensitivity of 97.98%, and positive predictivity of 97.55%.

Zheng et al. [53] developed an algorithm that precisely predicts the correct origins of idiopathic ventricular arrhythmia (IVA) and outperforms the accuracy of all prior studies found in literature and human experts. A total of 18612 ECG recordings extracted from 545 patients who underwent successful catheter ablation (CA) to treat idiopathic ventricular arrhythmia were proportionally sampled into training, validation, and testing cohorts. For it, was designed four classification schemes respond to different hierarchical levels of the possible IVA origins. For every classification scheme, 98 distinct machine learning models with optimized hyperparameter values obtained through extensive grid search were compared and reported an optimal algorithm with the highest accuracy scores attained on the testing cohorts. One of the developed machine learning-based ECG algorithms to predict 21 possible sites of IVA origin with an accuracy of 98.24% on a testing cohort. The accuracy and F1-score for the three left schemes surpassed 99%.

Che et al. [57] proposed an end-to-end deep learning framework based on a convolutional neural network for ECG signal processing and arrhythmia classification. The ECG signal data utilized in this study was acquired from a cardiology challenge, for which it had been collected from a total of 6877 individuals. Of these 6877 participants, 3178 were women, and 3699 were men, and each of these 12-Lead ECG recordings has a duration of between 6 and 60 s. A 7-layer CNN can automatically extract the relevant ECG features from these ECG signals. The method proposed was able to achieve an F1-score of 81.7% for the detection of normal beats, 85.8% for the detection of Atrial Fibrillation, 87.8% for the detection of First-degree atrioventricular block, 80% for the detection of Left Bundle Branch Block, 87.2% for the detection of Right Bundle Branch Block, 61.8% for the detection of Premature Atrial Contractions, 83% for the detection of premature ventricular contractions, 71.1% for the detection of ST-segment depression, 68.6% for the detection of ST-segment elevated, and an overall F1-score of 78.6%.

The authors of [65] introduce different ECG signal descriptors based on one-dimensional local binary pattern (LBP), wavelet, higher-order statistical (HOS), and morphological information for feature extraction, and a hybrid ECG arrhythmia classification approach called MRFO-SVM that combines a metaheuristic algorithm called Manta ray foraging optimization (MRFO) with support vector machine (SVM) for feature selection and classification. The database utilized in this study was the MIT-BIH arrhythmia database, which contains a total of 48 ECG files with a duration of 30 min each. The features extracted using the proposed morphological descriptors were the Distance between the R-peak and maximum amplitude in P duration, the distance between the R-peak and the two minimum amplitude values in QRS duration, and the distance between the R-peak and maximum amplitude in T duration. The extracted features of the R-R intervals found between consecutive beats are Pre-R-R, Post-R-R, Local-R-R, and Global-*r*-r. The MRFO-SVM method proposed by the authors achieved, when applied to the MIT-BIH arrhythmia database, an accuracy, sensitivity, specificity, precision, and F-score of 98.69%, 98.79%, 98.79%, 98.86%, and 98.97% respectively for beats belonging to the Normal beats class, an accuracy, sensitivity, specificity, precision and F-score of 98.20%, 98.20%, 99.20%, 96.45%, and 96.95% respectively for beats belonging to the Supraventricular Ectopic Beats class, an accuracy, sensitivity, specificity, precision and F-score of 98.05%, 98.05%, 99.83%, 99.38%, and 97.94% respectively for beats belonging to the Ventricular Ectopic beats class, and finally, an accuracy, sensitivity, specificity, precision and F-score of 96.50%, 96.50%, 99.59%, 96.05%, and 96.24% respectively for beats belonging to the Fusion class.

Hua et al. [66] proposed a framework based on neural networks to classify heartbeat arrhythmia problems. The model was trained with a Bayesian Neural Network (BNN) based on clinical features from the raw ECG data. The method was evaluated and compared between how the model makes decisions and what field experts do for the same problem. The main findings were that the features extracted from raw ECG data using the proposed framework could be used as an indication for specific heartbeat arrhythmia, the mechanism of the model can be interpreted as a decision-making procedure by weighing relative metrics just like human experts do, the weight of the features extracted from raw ECG data can be used to build a knowledge tree for guidance on diagnosing of specific heartbeat disease.

Li and Zhang [71] proposed a framing preprocessing method that can minimize the loss of ECG signals to enhance the features of signals and diagnose multiple types of cardiac arrhythmias. This study used the China Physiological Signal Challenge (CPSC) 2018 dataset comprising 6,877 (females: 3,178; males: 3,699) recordings of 12-lead ECG data collected from 11 hospitals. The proposed algorithm for classifying 12-lead ECG with multi-labeling consists of data denoising, framing blocking, dataset balance for data preprocessing, and a neural network structure based on ResNet in combination with attention-based bidirectional long short-term memory (BiLSTM). The developed algorithm was trained and tested on ECG data of nine types of cardiac states, fulfilling a multi-label classification task. It achieved an averaged F1-score and area under the curve at 0.908 and 0.974, respectively.

In [72], the authors developed a network model named Hybrid Convolutional Recurrent Neural Network for processing time series to predict multi-classification ECG. The database used was the MIT-BIH atrial fibrillation and contained 48 pieces of 2-lead ECG data, and each piece has 30 min. The features were extracted using the CNN method: the P, Q, R, S, T wave, and the R peak. The methods used were the long short-term memory, the gated recurrent unit, and the random forest. The method proposed has an accuracy of 99.01%, a sensitivity of 99.58%, a positive predictivity of 99.44%, and an F1-score of 99.51%.

In [74], the authors proposed an approach for detecting ventricular arrhythmias. This approach would transform ECG signals into images that could then be analyzed utilizing the AlexNet, VGG-16, and Inception-v3 deep learning models for feature extraction, with the actual classification of the beats being tested with several different classifiers. The authors extracted the ECG data utilized in this study from the MIT-BIH arrhythmia database, the Creighton University VT database, and Nsr. Three pre-trained CNN models were used for feature extraction in this study. The proposed method, after fusing Alexnet, VGG19, and Inceptionv3 DCNN features after entropy-based selection, was reported by the authors to achieve the best performance when using the cubic support vector machine as a final stage classifier, reaching a sensitivity of 98.2%, a specificity of 97.5%, a false negative rate of 2.3%, an accuracy of 97.6% and an F-score of 97.9%.

Nguyen et al. [76] proposed a method for single-lead ECG-based automatic recognition of atrial fibrillation by stacking a support vector machine on statistical features of segment-based recognition units produced by a convolutional neural network. The authors utilized the PhysioNet 2017 dataset to extract 8528 ECG signal recordings, of which 5076 were classified as normal beats, 758 as atrial fibrillation, 2415 as other beats, and 279 as noise. The authors then utilized the deep convolutional neural network to extract these signals' relevant features. From a total of 5 trials run by the authors, the average F1-score achieved by the proposed method was 84.19%.

Radhakrishnan et al. [77] proposed a time-frequency domain deep learning-based approach to detect atrial fibrillation and classify terminating and non-terminating atrial fibrillation episodes using ECG signals. In this study, two databases were used. The first database was Physionet Computing in Cardiology Challenge 2017 and contained 8482 single-lead ECG recordings with a sampling frequency of 300 Hz. The database consisted of 5156 normal recordings, 771 atrial fibrillation recordings, and 2557 records in the other rhythms classes. The second database is a mixture of the ECG recordings from the MIT-BIH atrial fibrillation dataset and the MIT-BIH arrhythmia dataset. The MIT-BIH AF dataset contains twenty-five records of two-lead ECGs with a sampling frequency of 250 Hz. The MIT-BIH arrhythmia dataset consists of forty-seven two-lead ECG recordings sampled at 360 Hz. The features extracted were the RR-interval, the QRS-complex morphology, and the P-wave. The proposed method, time-frequency representation, obtained an accuracy of 99.18%, a sensitivity of 99.17%, and a specificity of 99.18%.

The authors of [78] proposed a method for the automated classification of Ventricular Fibrillation and Ventricular Tachycardia using a deep neural network and hybrid time–frequency–based features. The authors utilized all the ECG records that made up the CUDB and the VFDB databases of the PhysioNet repository. The CUDB consisted of 35 8 min long records that, after being segmented into windows of 5 s, resulted in a total of 791 ventricular fibrillation windows and 2744 normal sinus rhythm windows. The VFDB consisted of 22 30 min long records that, after being segmented into 5-s windows, resulted in a total of 1202 ventricular tachycardia windows and 7618 normal sinus rhythm windows. The authors extracted several features from these ECG signals, like Filter Leakage Measure, Spectral Analysis (FSMN, A1, A2, and A3), Bandpass filter and auxiliary counts (C1 and C2), Covariance measure, Frequency calculation, Area calculation, Kurtosis, Standard Exponential Algorithm, Modified Exponential Algorithm, Skewness, Threshold crossing interval, Threshold crossing sample count, Hurst Parameter, Mean Absolute Value, Permutation Entropy, Non-linear Features such as Shannon entropy, Norm Entropy, Log Entropy, Threshold Entropy, and Sure Entropy. The proposed method achieved an accuracy of 99.2%, a sensitivity of 98.8%, and a specificity of 99.3%.

The authors of [86] proposed a robust and efficient 12-layer deep one-dimensional convolutional neural network for classifying the five micro-classes of heartbeat types in the MIT-BIH Arrhythmia Database. This database contained 48 ECG recordings. Each recording time was 30 min, the sampling frequency was 360Hz, and each ECG record was composed of two leads. In this study, the authors extracted features using convolution and pooling layers with two automatic extraction methods. The results obtained by the proposed CNN network method to the five micro-class classifications of heartbeats reach an accuracy of 97.41% and specificity and a positive prediction rate of over 90%.

The authors of [88] propose a classification method based on deep learning, namely, a global hybrid multi-scale convolutional neural network, to implement binary classification for Atrial Fibrillation detection using single-lead ECG recordings. The China Physiological Signal Challenge 2018 dataset (CPSC 2018) was used with a sample of 6877 12-Lead ECG recordings, of which 3699 belonged to males and 3178 to females. Of these 6877 recordings, 1098 (15.96%) were classified as suffering from Atrial Fibrillation, while the remaining 5779 (84.03%) were not. The authors used the GH-MS-CNN method to automatically capture and integrate the global discriminative multi-scale features related to atrial fibrillation patterns in all dense blocks layer-wise while ensuring relatively low computational cost. The authors reported an accuracy of 99.84%, a precision of 99.89%, a sensitivity of 99.65%, a specificity of 99.98%, and an F1-score of 99.54%.

In [89], the authors proposed an ECG-based arrhythmia classification method utilizing convolutional neural networks. The ECG data used by the authors during this study was extracted from the MIT-BIH arrhythmia dataset, which contains excerpts from 48-and-a-half-hour double-channel recordings extracted from 47 subjects. The convolutional neural network automatically handles the feature extraction. The results achieved by the proposed method averaged an accuracy of 99.76, a sensitivity of 94.45%, a specificity of 99.54%, and a positive predictive rate of 97.40%.

The authors of [90] proposed a 12-Lead ECG-based method for automatically classifying arrhythmias using a deep neural network. The authors got the ECG signal data for this study from the China Physiological Signal Challenge 2018 training database. The authors extracted two types of expert features from these signals, statistical features like mean, standard deviation, variance, and percentile, and Shannon entropy of signal processing features, extracted by applying discrete wavelet decomposition. The proposed method achieved, on average, a precision of 82.1%, a recall of 81.2%, an F1-score of 81.3%, an area under the receiver operating characteristic curve of 97%, and an accuracy of 96.6%.

In [92], the authors proposed an idea using a 14- layer deep learning model which consists of 1-Dimensional Convolutional Neural Networks (CNN) which extract representation features and Long Short-Term Memory (LSTM), which extract time sequence features that eventually pass into the Dense layer which classifies the ECG result into 4 classes. The classified result is then sent to the NodeMCU board, where the processing takes place, after which it is sent to Google Firebase. The result is stored in Firebase, which acts as a real-time database. The results and the notification are retrieved from the database, which is displayed in a mobile application. The Physionet Challenge 2017 CinC dataset has been used to build the deep learning model. The dataset houses 8528 samples of single-lead short ECG signals. The ECG signals have been sampled with a sampling frequency of 300Hz. The duration of each signal varies between the 20s and 1 min, reporting an overall F1 score of 81% obtained from the model.

The authors of [95] aimed to distinguish three arrhythmias types, A-Fib, AFL, and V-Fib, in ECG signals. The model proposed for R-peak automated detection was based on CNN applying dilated convolutions and residual connections. Also was introduced a model called Dilated CNN LSTM (DCNN-LSTM), to which the extracted segments around R-peaks were used as the input. The DCNN-LSTM final model consisted of dilated convolution layers and an LSTM layer to detect various arrhythmias. The performance of the proposed model on test samples was 98.93%, 99.78%, and 99.58%, respectively, in terms of overall accuracy, sensitivity, and specificity for tackling the problem of 4- class arrhythmia classification.

In [99], the authors presented an ML-based method for arrhythmia detection by extracting two important quantities, average energy and zero-crossing, as the features. ECG signals used in this study were obtained from 3 different databases, namely MIT-BIH arrhythmia, MIT-BIH normal sinus rhythm, and QT databases. SVM was utilized to classify the data containing two groups: normal and arrhythmia. 10-fold cross-validation for 10 min long ECG signals was the most suitable evaluating technique for the proposed method, with the respective performance results: 96.67% average accuracy, 93.33% sensitivity, 100% specificity, and 100% precision.

In [100], the authors proposed a method based on time domain features of ECG sequence and one-dimensional CNN to detect atrial fibrillation. The ECG data records were obtained from the MIT-BIH atrial fibrillation database, which contained 25 ECG signals, each with a recording time of 10h. The sampling frequency of the ECG signals was 250Hz, the resolution was 12bit, and the sampling bandwidth was 0.1–40Hz. The ECG signals were segmented into seven heartbeats, and 8 features were extracted based on the time domain features of the ECG sequence to form the feature vector (size 1*8). The convolutional neural network's one-hot label (1*2) output was combined with the extracted time domain features (size 1*8) to obtain 10-dimensional features. The extracted 10-dimensional features were normalized and then put into the SVM classifier. The experimental results showed that the proposed algorithm's sensitivity, specificity, and total accuracy were 99.07%, 97.05%, and 98.03%, respectively.

Hammad et al. [101] presented a deep neural network (DNN) strategy to determine appropriate information in ECG-based Arrhythmia diagnosis and treatment. The MIT-BIH database that contained 48 ECG records obtained from 47 subjects composed of 47% female and 53% male participants was used. It consisted of a learning stage where classification accuracy was improved via a robust feature extraction protocol. This is followed by a genetic algorithm (GA) to aggregate the best feature extraction and classification combination. Several classifiers were applied, including k-NN, support vector machine (SVM), and multilayer perception (MLP). A comparison of the performance recorded for the proposed technique alongside state-of-the-art methods reported that the area shows an increase of 94% and 95.3% in average accuracy and F1-score, respectively.

In [102], the authors investigated the impact of various data augmentation algorithms, e.g., oversampling, Gaussian Mixture Models (GMMs), and Generative Adversarial Networks (GANs), on solving the class imbalance problem in the classification of Atrial Fibrillation in Short Single-Lead ECG Signals. The data used in this study was obtained from the PhysioNet/CinC challenge 2017. It comprised 8528 single-lead ECG signals in four classes, namely 5154 Normal (N), 771 atrial fibrillation (AF), 2557 Noisy, and 46 Other rhythm signals. For data segmentation was chosen segment length SL = 1500 was equivalent to 5 s. The DCGAN Architecture was implemented. The ADAM optimizer with a learning rate of 10-3 and a decay rate of 10-5 was applied for training the deep neural networks. The ADAM optimizer with a learning rate of 10-3 and a decay rate of 10-5 was used for training the deep neural networks. On training the generator and the discriminator of the DCGAN, a learning rate equal to 2 × 10^−4^ was defined. As for the weight initialization in the network, the Xavier uniform initialization technique was applied. Among the augmentation algorithms investigated in this study, the lowest improvement in performance was achieved by oversampling, while GMM and DCGAN enhanced the performance the most. Although GMM marginally outperforms DCGAN in terms of the overall f1-score, it turns out that DCGAN results in better accuracy while producing comparable Normal class accuracy to GMM.

Hsu et al. [103] proposed a method for arrhythmia classification utilizing deep learning and machine learning, with features extracted by a waveform-based signal processing technique. The authors chose to extract the ECG data for this study from the MIT-BIH arrhythmia database, which contains records of 48 subjects, each with a duration of 30 min. Based on the waveform-based signal processing technique, the authors selected Gaussian and cubic spline features, which are automatically extracted. Of the machine learning methods utilized by the authors, the one that reaches the best results is the optimizable ensemble classifier, with an overall accuracy of 98.8%, and the deep learning-based classification method achieves an overall accuracy of 97.8%.

In [108] was developed a network of arrhythmia classification based on the CNN-Based Broad Learning System (CNNBLS). The ECG signals from the PhysioBank MIT-BIH arrhythmia database, which contained 48 groups of MLII and V5 leads at a 360 Hz sampling frequency, were used in this study. The proposed CNNBLS network extracted features through CNN and filtered out a part of the noise through convolution. The feature and enhancement nodes were taken as the extended input data, and the connection weights were approximated by ridge regression of the pseudoinverse. In addition, when CNNBLS encountered an incoming input. To compare the performance of the proposed system, traditional deep learning networks CNN and LSTM were implemented. The test accuracy of CNN and LSTM at 10,000 initial training sets was 98.56% and 97.34%, respectively, and the training time was 298.47 s and 175.22 s, respectively. When 12,929 incremental training data were added, the accuracy of CNN and LSTM was 98.93% and 97.87%, but the time spent on the training process was 696.95 s and 409.89 s, respectively. In original and denoising heartbeat data, the overall accuracy of CCBLS was 98.5% and 98%, respectively. When CNNBLS encountered incoming data, the test accuracy was increased to 98.45% with incremental learning, and the training time was only 47.23s.

Mazaheri et al. [112] proposed a method for diagnosing arrhythmias, which extracts morphological characteristics, frequency domain features, and nonlinear indices, reduces the feature space by utilizing metaheuristic optimization algorithms, and then uses machine learning algorithms, like k-nearest neighbor, feed-forward neural network, fitting neural network, radial basis function neural network and pattern recognition network, to classify the ECG signals. This study utilizes the MIT-BIH-Arrhythmia database, consisting of 48 dual-channel ECG registries from 45 patients. The method increased the performance of the classifier methods, with the best performing one, the feed-forward neural network, achieving an accuracy of 98.75%, a sensitivity of 98.84%, and a specificity of 99.85%.

Rahman et al. [113] proposed classification models based on classifying five classes of ECG arrhythmic signals from Physionet's MIT-BIH Arrhythmia Dataset. This dataset comprised 48 records of two channels of ECG signals for 30 min collected from 47 individuals. In this paper, a total of 109446 beats at 125 Hz sampling frequency from 44 records was evaluated as train and test patterns for the performance analysis of the 1D convolutional neural network model. Proposed CNN structure comprised 4 convolutional layers, three pooling layers afterward a single fully connected layer or dense layer, and a softmax. The model's overall accuracy was 95.2%, with an average precision and recall of 95.2% and 95.4%, respectively.

Subramanian and Prakash [114] analyzed heart diseases categorized as arrhythmia based on Electrocardiogram (ECG). ECG records from the MIT-BIH database of different disease conditions were investigated. The ECG signals were filtered to remove noise caused due to powerline interface or Electromyogram. This filtered signal was segmented into smaller pieces of ECG. The features extracted were Peak-to-peak Interval (R-R Interval), BPM (Beats per minute), and P-wave to QRS peak. The data set was classified using an SVM classifier algorithm. This algorithm classified the input ECG signal with varying feature parameters into two types of arrhythmias. This method achieved an accuracy of 91% with precision, recall, and an F1-score of about 0.906593.

In [115], the authors presented a dual fully-connected neural network model for the classification of heartbeats. The data utilized during this study was acquired from the Massachusetts Institute of Technology-Beth Israel Hospital arrhythmia database, containing 48 2-lead recordings from 47 individuals, of which 25 were males between the ages of 32 and 89 and 22 were females between the ages of 23 and 89. The MIT-BIH supraventricular arrhythmia database, consisting of 78 2-lead recordings, was also utilized for verification. The authors extracted a total of 105 features from these signals, like RR interval-related features: Anterior RR interval, Posterior RR interval, Local RR interval, Mean of RR interval, Normalized anterior RR interval, Normalized posterior RR interval, and Normalized local RR interval; Morphological features: Sampled QRS complex(10 points with 6-point intervals), a sampled neighborhood of T (8 points with 18-point intervals); Statistical features: Maximum, minimum, kurtosis, skewness, variance, mean, maximum to minimum ratio of heartbeats and QRS complex; Sum of trough features: Sum of the trough to describe the shape of the waveform; and Wavelet packet entropy: All the node energy at 6 levels. The overall accuracy of the proposed method was 93.4%, with a sensitivity and positive predictivity of 95.1% and 98.3%, respectively, for the detection of normal beats, sensitivity and positive predictivity of 90.3% and 43.5% for the detection of supraventricular ectopic beats, and sensitivity and positive predictivity of 84.1% and 89.5% respectively for the detection of ventricular ectopic beats.

In [119], the authors proposed an ECG-based multi-class Arrhythmia detection method using a Spatiotemporal attention-based convolutional recurrent neural network called STA-CRNN. The China Physiological Signal Challenge 2018 (CPSC 2018) database, containing 6877 12-Lead ECG records, is utilized as the training set for the proposed method, which is then evaluated using the private test set of CPSC 2018, containing 2954 12-Lead ECG records. A convolutional neural network automatically extracts the ECG features from the ECG signals in this database. The method proposed by the authors was able to achieve an F1-score of 81.9% for the detection of normal beats, 93.6% for the detection of atrial fibrillation, 86.6% for the detection of first-degree atrioventricular block, 86.2% for the detection of left bundle branch block, 92.6% for the detection of right bundle branch block, 78.9% for the detection of premature atrial contraction, 86.5% for the detection of premature ventricular contractions, 81.2% for the detection of ST-segment Depression, and 64% for the detection of ST-segment Elevated, with the average F1-score of the STA-CRNN method reaching 83.5%.

Bashar et al. [120] presented an automated and robust algorithm to detect Atrial Fibrillation using electrocardiogram (ECG) signals from ICU patients. Several statistical parameters were calculated from the heart rate, including root mean square of successive differences, Shannon entropy, Sample entropy, and turning point ratio. A subset of the Medical Information Mart for Intensive Care (MIMIC) III database containing 36 subjects were used in this study. Three classification algorithms were implemented and compared, including support vector machine (SVM), discriminant analysis (DA), and K-nearest neighbor (kNN). The results showed that the DA model achieved 99.80% sensitivity, 98.82% specificity, and 99.23% accuracy, kNN model with k-5, 99.76% sensitivity, 99.28% specificity, and 99.48% accuracy. Using the SVM classifier with radial basis kernel, the proposed method achieves 99.95% cross-validation accuracy on the training data and 99.88% sensitivity, 99.65% specificity, and 99.75% accuracy on the blinded test data.

Celin and Vasanth [122] developed a method of signal modeling based HRV signal approach to detect abnormality in ECG signals. The ECG signals were taken from the MIT-BIH arrhythmia database. In many cases of recordings, the upper signal was measured from ML II lead, and the lower signal was measured from V1 lead; V2, V5, and V4 leads were also used for several instances. FFT-based R-peak detection was proposed to extract the HRV signals. Next, the polynomial-based curve fitting was modeled. The statistical and wavelet parameters were used as features for the classification of normal and abnormal ECG signals using SVM, ANN, Adaboost, and Random Forests. The experimental result showed that the accuracy of the classifiers was 94.6%, 94.6%, 96.7%, and 98.8%, respectively.

The authors of [124] propose a method to detect arrhythmia in an imbalanced ECG dataset utilizing long short-term memory with focal loss. The MIT-BIH arrhythmia database provided the ECG signals used in this study. A total of 93371 ECG beats and eight beat types were considered for this study: Normal beat (N), LBBB (Left bundle branch block), RBBB (Right bundle branch block), APC (Atrial premature contraction), NESC (Nodal (junctional) escape beat), ABERR (Aberrated atrial premature beat), NPC (Nodal (junctional) premature beat), and AESC (Atrial escape beat). Of the 93371 beats, 75020 are N, 8072 are LBBB, 7255 are, 2546 are APC, 229 are NESC, 150 are ABERR, 83 are NPC, and 16 are AESC. A long short-term memory network automatically extracted the features from these recordings. Applying the proposed method to the dataset, the authors achieved an accuracy of 99.26%, a recall of 99.26%, a specificity of 99.14%, a precision of 99.13%, and an F1-score of 99.27%.

Kong et al. [126] proposed a machine learning method for rapid modeling and accurate diagnosis of atrial fibrillation. For this study, the electrical activity of the whole heart of the patients with atrial fibrillation and synchronous 12-Lead ECG signals was collected from atrial fibrillation patients and healthy people. The instrument used to manage the 12-Lead ECG signals was the Inno-12-U ECG. The dataset contains 1056 atrial fibrillation patients and 904 healthy people. The features were extracted using the Pan-Tompkins algorithm. The integrated radial basis function was combined with the relevance vector machine (IRBF-RVM). The results showed that the technique has a classification rate of over 97%.

In [127], the Physionet Computing in Cardiology Challenge 2017 data source was used for this study. The detection of the QRS complex, after which a completed analysis and delineation of each beat was obtained. In the preprocessing stage, the Discrete Wavelet Transform (DWT) was used for removing noise and tuning to the morphological characteristics of the waveform features. For feature extraction, a set of features that consists of both morphological and temporal features was extracted using DWT. A comparison study was conducted between five classifiers (Decision trees, Random Forest, AdaBoost ensemble classifier, support vector machine (SVM), and K-nearest neighbor Algorithm (KNN)) to evaluate the best diagnoses for each type of Arrhythmia. This study used four classes of coronary heart beats atrial fibrillation, normal, other rhythms, or noise. Results showed that the AdaBoost classifier gives 100% Accuracy scores for all types of Arrhythmias in the training set. The AdaBoost algorithm obtained a mean improvement report for all classes in the testing set (97.3% in Area under curve accuracy, 94.7% in classifier accuracy, 96.7% in sensitivity, and 98% in precision).

Nankani and Baruah [130] developed an end-to-end framework for classifying different length ECG segments into four classes: atrial fibrillation, normal, other, and noisy rhythms using a deep residual neural network, thereby eliminating the need for handcrafted features. A data augmentation technique was employed to make the model more robust toward the noise. The proposed method produces an F1 score of 0.88 ± 0.02 on the PhysioNet Computing in Cardiology Challenge 2017 database, consisting of four classes (recordings): Atrial Fibrillation (771), normal (5154), other (2557), and noisy (46) rhythms of lengths between 9 and 60 s sampled at 300Hz. The optimum length was between 3 and 7 s when the sampling frequency was kept and 7 s when the sampling frequency was around 300 Hz.

The authors of [131] proposed an 11-layer deep convolutional neural network model for classifying the MIT-BIH arrhythmia database into five classes according to the ANSI-AAMI standards. The database contained 48 subjects. The method used to extract features automatically was the CNN architecture. The proposed method used in this study was an accuracy of 98.3%.

The authors of [137] proposed a method for arrhythmia classification utilizing convolutional neural networks. The Massachusetts Institute of Technology-Beth Israel Hospital arrhythmia database, consisting of 48 lead II records collected from 47 individuals, of which 25 were male, ages 32–89, and 22 were female, ages 23–89, was utilized by the authors as this study's source of ECG signals. The extraction of features from these ECG signals is handled automatically by the convolutional neural network. The proposed method performs at a high level for arrhythmia detection, reaching an accuracy of 99.06%.

Wang et al. [138] proposed a CNN-based method for multi-class arrhythmia detection with multiple-stage features fusion. The significant contributions of this study were as follows: connection operations to fuse different levels of features extracted by the neural network at various stages for target task processing were skipped. And the channel-wise attention modules were adopted to extract the features learned at the different stages. By combining the attention module and convolutional neural network, the discrimination power of the network for ECG classification was improved. The proposed model was validated on the China Physiological Signal Challenge 2018 database and demonstrated its performance in classifying 9 cardiac arrhythmias using 12-lead ECG signals. The proposed method for ECG classification was compared on an open ECG dataset with some state-of-the-art methods, which achieved an average F1-score of 81.3%.

Abdeldayem and Bourlai [142] investigated the classification of cardiac arrhythmia using the patient's ECG signal by assessing three algorithms. A temporal texture-based algorithm was utilized, which extracts the 1D-LBP features of the ECG signal. The integration of the second ECG lead (i.e., using both ECG lead configurations) has boosted the system's accuracy by an average of 10% since additional information is gained compared to using only one lead. On the other hand, spectro-temporal STFT texture features provided more knowledge than only temporal information since it reflects the variation of frequency components over time, where a boost of 7% was achieved. The CWT has an overall performance improvement, especially on the sub-class level, with an average 15% increase in the F-score. The SVM classifier has proven its superiority compared to KNN, which suffered from over-fitting when using a 10 cross-validation on the MIT-BIH database. The proposed system achieved an accuracy of 99.81% in the time-frequency domain using an SVM classifier and an input ECG signal of 0.56 s.

Ebrahimzadeh et al. [143] aimed to address a validated method to predict the onset of Paroxysmal Atrial Fibrillation (PAF) by integrating classical and modern techniques. In this study, 106 data from 53 pairs of ECG recordings were obtained from the standard database called Atrial Fibrillation Prediction Database (AFPDB). Each ECG segment contains a recording with a sampling rate of 128 Hz and 12-bit resolution. The features the authors extracted from the ECG recordings were the HRV signal, the time-domain features, and the frequency-domain features. The method used was the local subset feature selection. The authors used 2 classifiers, the Support Vector Machine and the K-Nearest Neighbor, to help the proposed method. The first classifier has an accuracy of 94.64%, a specificity of 93.10%, a sensitivity of 96.29%, and a precision of 92.85%. The second has an accuracy of 89.28%, a specificity of 86.66%, a sensitivity of 92.30%, and a precision of 85.71%.

The authors of [145] combined several data mining techniques, such as clustering, feature selection, oversampling strategies, and automatic classification algorithms, to create more efficient arrhythmia classification models. The dataset utilized during this study was acquired from the machine learning repository UCI and had 280 attributes. The authors used CfsSubsetEval as the method for automatic extraction of the ECG features from these signals. Analyzing the impact of combining these data mining techniques with classification algorithms, like Random Forest classifiers, the authors proved it increased their accuracy and Macro-F1 value. In the specific case of the Random Forest classifier, the Macro-F1 value of the algorithm increased consistently from its original value of 62.3% to its 72.7% value when applying feature selection, to 81.9% when applying classification using local clustering with oversampling, and finally to 88.8% when applying both feature selection and classification using local clustering with oversampling.

The authors of [151] propose the representation of electrocardiogram signals using sparse decomposition over the composite dictionary to recognize cardiac arrhythmias automatically. The ECG data utilized during this study was collected from the MIT-BIH arrhythmia database, which contains 48 30-min records from 47 patients. The authors extracted five features from these ECG signals: permutation entropy, energy, RR-interval, standard deviation, and kurtosis. Having applied the proposed method to the database, the authors report an overall accuracy of 99.21%, an overall sensitivity of 99.21%, and an overall F-score of 99.21%.

In [152], the authors proposed a feature extraction method using the sparse representation technique to efficiently represent the different ECG signals for efficient analysis of cardiac arrhythmias. The database used was MIT-BIH arrhythmia, including 48 2-Lead ECG recordings of patient data. The data were sampled at a rate of 360 Hz. The ECG features were extracted using machine learning techniques, SVM, K-NN, PNN, and RBFNN, and optimized using ABC and PSO techniques. The best method to classify the ECG signals was the LSTSVM + PSO, with an accuracy of 89.93%, a sensitivity of 91.47%, and a positive predictivity of 85.88%.

Warrick et al. [153] proposed an algorithm combining a Convolutional Neural Network and a sequence of Long Short-Term Memory units to identify cardiac arrhythmias and noise in electrocardiograms. The authors utilized the ECG signals provided for the PhysioNet/CinC Challenge 2017, which contained 8528 signals. Of these 8528 signals, 5076 presented normal sinus rhythm (N) cases, 758 atrial fibrillation (AF) cases, 2415 alternative rhythms (O), and 279 noisy cases. The relevant ECG features were automatically extracted for each of these entries using a one-layer CNN. The study's authors reported that the method achieved an overall F1-score of 82.0%, with an F1-score for N of 90.28%, for AF of 82.21%, and an F1-score for O of 73.24%.

Wu et al. [154] proposed an end-to-end model for generic and personalized ECG arrhythmic heartbeat detection on ECG data from wearable and non-wearable devices. Firstly, a deep learning-based model was developed to address the challenging problem caused by inter-patient differences in ECG signal patterns. This model achieves state-of-the-art performance for ECG heartbeat arrhythmia detection on the commonly used benchmark dataset from the MIT-BIH Arrhythmia Database. Then the model was used in an active learning process to perform patient adaptive heartbeat classification tasks on the non-wearable ECG dataset from the MIT-BIH Arrhythmia Database and the wearable ECG dataset from the DeepQ Arrhythmia Database. Results showed that our personalization model requires a query of less than 5% of data from each patient, significantly improves the precision of disease detection from the generic model on each subject, and reaches nearly 100% accuracy in normal and VEB beat predictions on both databases.

In [156], the authors developed a 12-layer deep convolutional neural network to train a classification model to distinguish between Atrial Fibrillation and non-Atrial Fibrillation cases. For this study, the authors utilized ECG signals from the MIT-BIH AF database, consisting of 25 recordings from 25 patients every 10 h and 15 min in duration. The authors automatically extract the relevant ECG features from the signals in this database using the convolutional neural network, with no need for manual intervention. Utilizing the MIT-BIH Atrial Fibrillation Database and excluding an inferior signal quality ECG recording in the test data, the method proposed by the authors in this study was able to achieve a mean accuracy of 84.85%, a mean sensitivity of 79.05%, a mean specificity of 89.99% and a result for the area under the receiver operating characteristic curve of 0.92.

Zhang et al. [157] proposed a nine-layer convolutional neural network (CNN) that can automatically extract appropriate features and detect the different categories of ECG beats based on individual records. A ten-fold cross-validation strategy was designed that boosts the robustness of our proposed CNN model. The dataset consisted of three types of ECG beats, namely: normal beat (N), premature ventricular contraction beat (V), and right bundle branch block beat (R). The experimental results demonstrated that with an appropriate choice of structure and parameters, the proposed deep learning model could classify the ECG hearts with a sensitivity of 98.37%, specificity of 99.19%, and accuracy of 98.92%.

Andreotti et al. [159] classified segments of ECG into four classes (AF, normal, other rhythms, or noise) as part of the Physionet/Computing in Cardiology Challenge 2017. It was compared with a state-of-the-art feature-based classifier with a convolutional neural network approach. Both methods were trained using the challenge data, supplemented with an additional database derived from Physionet. The feature-based classifier obtained an F1 score of 72.0% on the training set (5-fold cross-validation) and 79% on the hidden test set. Similarly, the convolutional neural network scored 72.1% on the augmented database and 83% on the test set. The latter method resulted in the competition's final score of 79%.

In [160], the authors propose an algorithm for Atrial Fibrillation detection based on the irregularity of heart rate, the absence of P-waves, and the presence of fibrillatory waves, in which the Support Vector Machine classification model makes the distinction between Atrial Fibrillation and non-Atrial Fibrillation episodes. The ECG signals utilized in this study were acquired from 12 different patients, with the data from one of them coming from the St-Petersburg Institute of Cardiological Technics 12-lead Arrhythmia Database and the data from the other 11 coming from the Cardiorisk - Personalized Cardiovascular Risk Assessment through Fusion of Current Risk Tools project. The authors determined the most relevant features for the detection of Atrial Fibrillation episodes based on the F-scores these were able to achieve when utilized in the tests and ended up with a total of eight ECG features. The method proposed by the authors in this study for the detection of Atrial Fibrillation in 12-Lead ECG signals, when applied to the database utilized in this study, was able to reach a sensitivity of 88.5%, a specificity of 92.9% and a positive predictive value of 90.6%.

Khatun and Morshed [162] explored the robust detection of both Myocardial Infarction and Arrhythmia from ECG signals using minimalistic (single-channel) ECG data. This study used 440 records from 12-lead ECG signals (79 Healthy Person, 346 MI, and 15 AR) from the “PTB Diagnostic ECG Database” of PhysioBank. The proposed algorithm automatically identified the ECG signals' P, Q, R, S, and T points and then extracted 33 features: 15 interval type and 18 amplitude type. Bagging Tree, a computationally efficient ensemble method that can deal with the class imbalance problem within the data, was used for classification. During training, 10-fold cross-validation was used to ensure the classifier's generalization and to remove over-fitting. This study demonstrated that Bagging Tree was able to identify MI, AR, and normal patients with a cross-validation accuracy of 99.7%, a sensitivity of 99.4%, a specificity of above 99.5%, a precision of 99.32%, an F1-score of 99.36% from a single lead ECG data (Lead V4).

In [165], the authors proposed an autonomous and robust method of distinguishing between pathological and normal recordings. The presented method consisted of the following blocks – signal transformation (primarily to envelograms), QRS detection, signal averaging, feature extraction, and their processing with machine learning (ML) and simple logical rules. The data processing was as follows: an ECG file (1 lead, 300 Hz sampling, AliveCor device) was loaded and transformed into envelograms intended for QRS detection (LF: 1–8 Hz, MF: 5–25 Hz, HF: 45–65 Hz) and a convolution neural network (1–5 Hz, 5–10 Hz, etc. up to 35–40 Hz). The 120 most important features and outputs from the neural network were fed into a bagged tree ensemble. Machine-learning algorithms and logical rules were trained using 8,138 files from a reduced training set. The F1-score measured using a hidden test set (3,658 recordings) was 81%.

Shimpi et al. [166] used 4 classifiers for the classification of cardiac arrhythmia, namely: Random Forest Algorithm, Support Vector Machine, Logistic Regression, and KNN classifier. When the dataset was cross-validated and tested, the maximum accuracy was obtained by the Support Vector Machine Classifier. The accuracy obtained was 91.2%.

Soliński et al. [168] proposed an algorithm for Atrial Fibrillation and other arrhythmias classification of short-term single lead ECG signals, which was the aim of the PhysioNet Challenge 2017. The database comprises over 8.5 thousand ECG recordings (between 10 and 60 s length) measured by the AliveCor device. An alternative hybrid approach for QRS detection was prepared to obtain RR time intervals. It consists of two complementary methods in hierarchical order: one based on nonlinear transformation and the first-order Gaussian differentiator as superior and another proposed in sample entry as inferior. Was introduced the machine learning algorithm to classify whether it is a normal sinus rhythm, Atrial Fibrillation, or an alternative heart rhythm using features considered regularity of RR time intervals and morphology of the ECG signal. The separate part of the algorithm based on the beat averaging method was dedicated to the preceding extraction of too noisy recordings from the input to the classifier. The best overall F1-score achieved in the official phase of the PhysioNet Challenge 2017 was 77%.

In [170], the authors introduced an approach to automatically detect and classify cardiac arrhythmias in electrocardiogram (ECG) recordings. The proposed method used a combination of Convolution Neural Networks (CNN) and a sequence of Long Short-Term Memory (LSTM) units, with pooling, dropout, and normalization techniques to improve their accuracy. The network predicted a classification at every 18th input sample and selected the final prediction. Results were cross-validated on the Physionet Challenge 2017 training dataset, which contained 8,528 single lead ECG recordings lasting from 9s to just over the 60s. Using the proposed structure and no explicit feature selection, 10-fold stratified cross-validation gave an overall F-measure of 0.83.10 ± 0.015 on the held-out test data (mean ± standard deviation over all folds) and 0.80 on the hidden dataset of the Challenge entry server.

### 3.3 Myocardial dysfunction/pathologies

Mazidi et al. [50] proposed a detection system for premature ventricular contraction (PVC) based on the tunable Q-factor wavelet transform algorithm and statistical methods to detect PVC. The authors extracted 22 ECG records from the MIT-BIH arrhythmia database for this study. The tunable Q-factor wavelet transform algorithm handles the feature extraction in this paper. The authors extracted the QRS width and R peaks and, in a second step, nine features from these ECG signals: minimum, maximum, root mean square, mean, interquartile range, standard deviation, skewness, and variance. The system then uses three machine learning classifiers, Support Vector Machine, K-Nearest Neighbor, and artificial neural network, to evaluate the extracted features. The best results were achieved with the K-Nearest Neighbor algorithm, reaching a sensitivity of 98.23% and an accuracy of 97.81%.

Dey et al. [54] proposed unique architecture that systematically processes 12-lead ECGs based on employing handcrafted features to discriminate the multiple classes, resulting in the development of o a detection model consisting of a one-dimensional (1-D) convolutional neural network (CNN) and a bidirectional long short-term memory (bi-LSTM) layer which classifies into three classes, namely: healthy control (HC), Myocardial Infarction (MI), and non-myocardial infarction (non-MI) subjects for a realistic and reliable assessment. The model's performance was evaluated using 517 records acquired from the Physikalisch-Technische Bundesanstalt (PTB) database with an accuracy of 99.246%, kappa of 0.983, and macro averaged F1-score of 98.86% achieved using stratified 5-fold cross-validation.

Wang et al. [82] proposed an improved gated recurrent unit by setting a scale parameter into the existing bidirectional gated recurrent unit (BGRU) model for premature ventricular contraction signals recognition. To verify the effectiveness, the IGRU model was embedded into a convolutional network frame, and existing GRU and BGRU models were employed as control groups for a fair comparison. The databases used were the MIT-BIH arrhythmia database and China Physiological Signal Challenge 2018. The first database consisted of 48 records. The second consists of 6877 records with a sampling rate of 500 Hz and a duration of 6s–60s. For data consistency, the authors downsampled the ECG records in both databases to 250 Hz and divided them into 2s episodes. All the features used in this study were automatically extracted. The proposed method, CNN-IGRU, performs for the first database with an accuracy of 98.3%, a sensitivity of 98.4%, and a specificity of 98.2%. For the second database, an accuracy of 97.9%, a sensitivity of 98%, and a specificity of 97.8%.

Xiong et al. [87] developed a multi-lead myocardial infarction localization approach based on the dense convolutional network (DenseNet). The 12-Lead ECG data was from Physikalisch-Technische-Bundesanstalt (PTB). This study selected 448 records, specifically 80 records from 52 healthy subjects called healthy control and 368 records from 148 myocardial infarction patients. The features were extracted using the DenseNet method, and the Pan Tompkins algorithm detected the R peak. Beyond the DenseNet method, the convolutional neural network method was used. The proposed method achieved an accuracy of 99.87%, a sensitivity of 99.84%, and a specificity of 99.98%.

Ibrahim et al. [105] utilized the ECG-ViEW II database to propose three machine learning models to predict Acute Myocardial Infarction (AMI) risk conditions. ECG-ViEW II contained 979,273 extracted ECG measurements and other information regarding diagnoses, drug prescriptions, and selected laboratory test results collected from 371,401 patients over 19 years. Three proposed predictive models showed promising results when evaluated across all 5-performance metrics. The RNN model underperformed compared to the CNN model due to its more fitting application to time-series data and not static data. The CNN model shows a minimum F1-score of 89%, minimum sensitivity of 88%, and minimum specificity of 93% beating many states of-the-art literature approaches. The best model was the XGBoost model, with an F1 score of 97.1%, a sensitivity of 93.5%, and a specificity of 99.4%. Due to the tabular nature of the dataset. Testing the CNN and RNN models without the age and sex features a reduced performance by an average of 3.78% and 5.9% across the 5 metrics, respectively. Shapley value analysis shows that age, ACCI, and QRS duration are the most crucial variables in predicting the onset of AMI.

Prabhakararao and Dandapat [111] proposed a method for detecting Myocardial Infarction (MI) patients from non-MI patients and health control HC. Each lead of the incoming 12-lead ECG was first fed to the respective weights shared temporal encoding (TE) block to capture the lead-specific temporal dependencies. An intra-lead attention module corresponding to each lead was developed to focus on the most discriminative ECG features to obtain a lead-specific attentive representation (LSAR). An inter-lead attention module combines these representations across the 12-leads to form an inter-lead attentive representation. This representation was concatenated with the five PC features (c ∈ R5) and fed to a standard Softmax classifier for the classification. The present generalizable classification results, a patient-independent 5-fold cross-validation technique, resulted in an overall accuracy of 98.3%.

The authors of [116] proposed a method for automated detection of myocardial infarction utilizing multi-feature fusion and random forests. The ECG data used during this study was extracted from the PTB MI ECG database (Goldberger et al., 2000), which consists of 549 records collected from 290 subjects, 209 of which were males with a mean age of 55.5 years and 81 of which were females with a mean age of 61.6. From these signals, the authors extracted statistical (mean, standard deviation, skewness coefficient, and kurtosis coefficient) and entropy features (signal entropy, Shannon entropy, Renyi entropy, and Tsallis entropy) as the representation of the first layer features for each lead and extracted second layers features resorting to the use of random forests. The authors then employed 2 schemes for the intra-patient and inter-patient to evaluate the proposed method. For the intra-patient scheme, the technique achieved an accuracy of 99.71%, a sensitivity of 99.7%, a specificity of 99.73%, and an F1-score of 99.71%. The inter-patient scheme attained an accuracy of 85.82%, a sensitivity of 73.91%, a specificity of 97.73%, and an F1-score of 83.9%.

Boppana et al. [121] proposed the combined format of Clustered based K-medoid and Classification based K-NN along with hyperparameter tuning to classify abnormalities in ECG, mainly focusing on Myocardial infarction (MI) and Obstructive Sleep Apnea (OSA). ECG Data capturing System by using machine perception, digitization of ECG image data was considered and converted into binary image format to remove ambient noises and disruptions and extract the features from binary image format using wavelet transforms. AR models and their coefficients were combined and concatenated to form a 1-D vector where the calculated intervals and amplitude features were stored in that vector. The pre-processing process of ECG image based on clustering uses K-medoid last performance was evaluated using KNN and hyperparameter. KNN showed an accuracy of 86% in the existing system, and to detect OSA and MI from ECG abnormalities where k = 5, which was given an accuracy of 90%.

Prabhakararao and Dandapat [132] presented the Posterior Myocardial Infarction (PMI) detection algorithm from health control (HC) subjects using 3-lead ECG signals. The proposed method consisted of four stages, such as VCG signal preprocessing to reduce BW and HF noises, multiscale sub-band matrices (MSSM) were constructed from preprocessed ECG signal using 6-level wavelet decomposition, covariance structures of selected MSSM to obtain a 12-dimensional MSEF for efficient classification, and supervised binary classification of 3-lead ECG signals as HC or PMI using weighted SVM classifier to combat data imbalance problem. The publicly available PhysioNet/PTBDB diagnostic database validated the proposed method using 1463 HC and 148 PMI 4 sec 3-lead ECG signals. The best test accuracy of 96.69%, the sensitivity of 80%, and the Gmean of 88.72% were achieved by a 12- dimensional MSEF and weighted SVM-RBF classifier.

In [139], the authors combined random forests and ECG data to develop an ECG left ventricular hypertrophy classifier. For the ECG data to meet the requirements of the random forests, this study also proposes a method that uses K-nearest neighbors imputes missing values of ECG data and Z-score to standardize ECG data. The data utilized in this study was obtained from the Cardiovascular Disease Risk Factors Two-Township Study, from which the authors could acquire recordings from 767 subjects. Of these 767 people, 385 were female, and 382 were male. The female subjects had a mean age of 67, while the male subjects had a mean age of 68, and the mean BMI of both groups was the same at 24. A total of 327 patients had left ventricular hypertrophy, with 206 (53.5%) female participants and 121 (31.6%) male participants. From these ECG signals, the authors extracted P waves representing atrial depolarization, PR intervals, QRS complexes representing ventricular depolarization, T waves representing ventricular repolarization, and QT intervals representing the duration of left and right ventricle depolarization and repolarization. The results of the application of this method vary depending on the number of decision trees utilized, with 50 being the number that, in the tests run by the authors, resulted in the best outcomes, with a sensitivity of 58.4%, a specificity of 70.9% and an accuracy of 66.1%.

Iqbal et al. [148] proposed a method for recognizing myocardial infarctions and atrial fibrillation patterns, called deep deterministic learning, combining predefined heart activities with fused datasets. Two datasets were used during this study, the Massachusetts Institute of Technology–Beth Israel Hospital dataset and one provided by the University of Malaya Medical Center. From these signals, the authors extracted seven-time features, including heart rate, mean of HR, the standard deviation of RR interval, root mean square difference of RR interval, number of RR intervals more than 50, the mean value of T-wave onset, and mean value of the T-wave offset. The proposed method achieved an overall accuracy of 99.88% for detecting myocardial infarction, 100% for detecting normal sinus rhythms, and 99.97% for detecting atrial fibrillation.

The authors of [164] presented an innovative genetic ensemble of classifiers for classifying cardiac disorders based on electrocardiography signal analysis. The ECG signals were obtained from the PhysioNet service from the MIT-BIH Arrhythmia Database. The database has 1-lead ECG signals from 29 patients, and each signal contains 17 classes: normal sinus rhythm, pacemaker rhythm, and 15 cardiac dysfunctions. All ECG signals were recorded at 360 Hz. The features were extracted using the Welsh method. The method used was the support vector machine (SVM). Ensembles proposed method in this study was an accuracy of 91.40%, a sensitivity of 91.40%, and a specificity of 99.45%.

### 3.4 Other cardiovascular pathologies

In [23], the authors proposed a fast and accurate classifier that simulates the cardiologist's diagnosis to classify the ECG signals into normal and abnormal from a single-lead ECG signal. The features extracted were the R, P, Q, S, and T peaks, the T wave, the P-R interval, the R-R interval, and the S-T interval. The proposed method approaches an accuracy of 99%.

The authors of [22] presented a deep learning-based diagnosis system for the early detection of heart failure, particularly in elderly patients. The databases used in this study were the MIMIC-III, containing 61532 patients' data, and the physiological waveform database, containing 10282 patients’ data. The features were extracted automatically using the CNN method. The other method used in this study was the 33. The results for this last method were an accuracy of 97.5%, a sensitivity of 97.7%, and a specificity of 97.4%.

Anand et al. [39] implemented the ST–CNN–GAP-5 model on a publicly available PTB-XL ECG signal dataset to detect cardiac disorders. This dataset is available on Physionet, and the ECG data was recorded from 1989 to 1996. It's a 12-Lead ECG dataset consisting of 21,837 samples from 18,885 patients aged between 1 and 95 years, where gender is equally balanced, with 52% male and 48% female. For the proposed study, the authors extracted the QRS complex representing ventricular depolarization, the ST-T-U complex (ST segment, T, and U) representing ventricular repolarization, and the P-wave representing atrial depolarization. Using this method, the authors generated two groups of results, one at 500 Hz and one at 100 Hz. The first group has an accuracy of 95.85%, a Macro AUC of 99.46%, a Macro AUPRC of 98.53%, a Macro Precision of 95.44%, a Weighted of 95.85%, a Macro Recall of 95.34%, a Weighted Recall of 95.85%, a Macro F1 of 95.39%, and a Weighted F1 of 95.84%; the second group has an accuracy of 96.22%, a Macro AUC of 99.54%, a Macro AUPRC of 98.73%, a Macro Precision of 95.90%, a Weighted of 96.25%, a Macro Recall of 95.71%, a Weighted Recall of 96.22%, a Macro F1 of 95.79%, and a Weighted F1 of 96.22%. Based on these results, the performance at 100 Hz is better than that at 500 Hz.

Guo et al. [42] developed a pragmatic prediction model based on T wave inversion (TWI) and S wave in lead V1 (SV1), which can automatically be acquired by electrocardiography to screen for Hypertrophic cardiomyopathy (HCM). All enrolled participants had data from at least one standard 12-lead ECG and transthoracic cardiac echocardiography examination. Model development was performed according to the Transparent Reporting of a Multivariable Prediction Model for Individual Prognosis or Diagnosis (TRIPOD) guidance. Four different approaches were applied: (1) the adaptive least absolute shrinkage and selection operator (LASSO) analysis; (2) LASSO followed by multivariable logistic regression with backward stepwise selection; (3) LASSO followed by best subset selection; and (4) multivariable logistic regression with backward stepwise selection. After several independent feature selection approaches and model evaluation, only two ECG features were included, T wave inversion (TWI) and the amplitude of S wave in lead V1 (SV1) in the HCM prediction model. The model showed a useful discriminative performance (C-statistic >0.75) in training [C-statistic 0.857 (0.818–0.896)] and temporal validation cohorts [C-statistic 0.871 (0.812–0.930)]. In the external validation cohort, the C-statistic of the model was 0.833 [0.825–0.841].

In [51], a deep learning model to detect significant aortic regurgitation using ECG is proposed. The dataset utilized during this study consisted of 29859 ECG–echocardiography pairs, including 412 AR cases from 170 patients, and was collected using a 12-Lead ECG sensor, model FCP-8700/FCP-8800 by Fukuda Denshi. The mean age of the study population was 63.3 ± 16.9 years, and of the 29859 ECG–echocardiography pairs, 16922 12-Lead ECGs were from 9010 men and 12,937 12-Lead ECGs from 7334 women. For the study, nine features from the automatic 12-Lead ECG analysis results were considered: heart rate, presence of atrial fibrillation, RR interval, PR interval, QRS duration, QT interval, corrected QT interval, QRS axis, and P-wave axis. Two demographic variables (age and sex) were also collected from the record when the 12-Lead ECG was performed. The authors developed a multi-input neural network model consisting of a two-dimensional convolutional neural network (2D-CNN) using raw ECG data and a YB (FC-DNN) using ECG features. The results achieved when applying this method prove that it can detect significant aortic regurgitation with modest predictive value: 82.3% accuracy, 53.5% sensitivity, 82.8% specificity, 99.1% negative predictive value, and 5% positive predictive value.

Zhao et al. [52] developed a deep learning model based on the convolutional neural network long short-term memory (CNN-LSTM) to detect left ventricular hypertrophy (LVH) using 12-lead ECG. The echocardiogram and ECG of 1,863 patients obtained within one week after hospital admission were analyzed. Patients were evenly allocated into 3 sets at a 3:1:1 ratio: the training set (n = 1,120), the validation set (n = 371), and the test set 1 (n = 372). In addition, we recruited 453 hospitalized patients into the internal test set 2. Different DL model of each subgroup was developed according to gender and relative wall thickness. The LVH was predicted by the CNN-LSTM model with an area under the curve (AUC) of 0.62 (sensitivity 68%, specificity 57%) in the test set 1, which outperformed Cornell voltage criteria (AUC: 0.57, sensitivity 48%, specificity 72%) and Sokolow-Lyon voltage (AUC: 0.51, sensitivity 14%, specificity 96%). In the internal test set 2, the CNN-LSTM model had a stable performance in predicting LVH with an AUC of 0.59 (sensitivity 65%, specificity 57%). In the subgroup analysis, the CNN-LSTM model predicted LVH by 12-lead ECG with an AUC of 0.66 (sensitivity 72%, specificity 60%) for male patients, which performed better than that for female patients (AUC: 0.59, sensitivity 50%, specificity 71%).

The authors of [58] propose a deep convolutional network for classifying cardiovascular diseases utilizing standard 12-Lead ECG signals. The ECG data used in this study was acquired from the PTB Diagnostic ECG Database. This database has a total of 549 records obtained from 290 subjects, of which 209 were male, and 81 were female, ranging from 17 to 87 years old. Of the 549 records, this database has 148 myocardial infarctions, 52 normal beats, 7 hypertrophic cardiomyopathies, 6 dilated cardiomyopathies, 14 bundle branch blocks, and 6 valvular heart diseases. To increase the amount of data the authors had to work with, they first divided the database into intervals of 1, 2, and 3 s. The convolutional neural network automatically takes care of the feature extraction and feature selection in this study, removing the need for expert knowledge. Applying this method to the database, the authors were able to achieve accuracy, sensitivity, and specificity of 99.59%, 99.04%, and 99.87%, respectively, with 1-s ECG signals, an accuracy, sensitivity, and specificity obtained of 99.80%, 99.48%, and 99.93%, respectively, using 2-s of signals with pre-trained proposed models, and accuracy, sensitivity, and specificity of segmented ECG tested by 3-s signals of 99.84%, 99.52%, and 99.95%, respectively.

The authors of [59] proposed a 12-Lead ECG-based cardiac amyloidosis detection method utilizing artificial intelligence. The authors collected the ECG data used in this study from a group of 2541 patients, with the inclusion criteria being a diagnosis of Transthyretin Amyloidosis or light chain-associated amyloid in the last 180 days. The AI automatically handles the feature extraction in this study. The method proposed achieved an area under the receiver operating characteristic curve of 0.91, with a positive predictive value for detecting Transthyretin Amyloidosis or light chain-associated amyloid of 86%. The AI was also able to predict the presence of these conditions in patients subjected to ECG tests pre-diagnosis, more than 6 months before the clinical diagnosis, on 59% of occasions. The best single-lead model was V5, with an area under the receiver operating characteristic curve of 0.86 and a precision of 78%. The 6-lead model had an area under the receiver operating characteristic curve of 0.90 and a precision of 85%

Haleem et al. [60] proposed a two-stage multiclass algorithm. The first step performs ECG segmentation based on convolutional bidirectional long-term memory neural networks with attentional mechanisms. A second stage was based on a time-adaptive convolutional neural network applied to ECG beats extracted from the first stage for various time intervals. The authors used one dataset for training/testing the ECG segmentation model and four datasets for the cardiovascular disease model detector for training/testing. The first dataset was trained, validated, and tested using PhysioNet's QT dataset and contained 105 2-lead ambulatory ECG recordings with P, QRS, and T waves. The other four datasets were: MIT-BIH Normal Sinus Rhythm Database, containing 18 long-term ECG recordings of normal healthy not-arrhythmic subjects (thirteen females between 20 and 50 years old); BIDMC Congestive Heart Failure Database, including 15 long-term ECG recordings of subjects with severe CHF (eight females between 22 and 63 years old); the MIT-BIH Sudden Cardiac Death Holter database, containing 18 patients with sustained ventricular tachyarrhythmia (eight females between 17 and 89 years old); and the last one was MIT-BIH Arrhythmia Database obtained from 47 subjects (22 females between 23 and 89 years old). The features extracted were P, QRS, and T waves. The CVD method achieved 100% accuracy in detecting events, such as normal sinus rhythm, congestive heart failure, arrhythmia, and sudden cardiac death, based on different time intervals of the ECG recordings.

In [68], the authors developed an automated system for automatically categorizing electrocardiogram signals into normal coronary artery disease, myocardial infarction, and congestive heart failure classes using a convolutional neural network and unique GaborCNN models. This study utilized Fantasia and St. Petersburg databases containing 2-lead ECG signals from 92 healthy controls, 7 coronary artery disease, 148 myocardial infarctions, and 15 congestive heart failure patients. The authors used the CNN model for automated extraction. The methods used were CNN and GaborCNN. For the CNN method, the results were: the normal class was a sensitivity of 98.85%, a specificity of 99.49%, a positive predictive value of 99.60%, and an accuracy of 99.13%; the myocardial infarction class was a sensitivity of 99.95%, a specificity of 99.95%, a positive predictive value of 99.58%, and an accuracy of 99.95%; the coronary artery disease class was a sensitivity of 98.67%, a specificity of 99.35%, a positive predictive value of 95.96%, and an accuracy of 99.26%; the congestive heart failure class was a sensitivity of 99.64%, a specificity of 99.90%, a positive predictive value of 99.62%, and an accuracy of 99.85%. For the GaborCNN method, the results were: the normal class was a sensitivity of 97.95%, a specificity of 99.39%, a positive predictive value of 99.52%, and an accuracy of 98.58%; the myocardial infarction class was a sensitivity of 99.13%, a specificity of 99.75%, a positive predictive value of 97.82%, and an accuracy of 99.68%; the coronary artery disease class was a sensitivity of 98.56%, a specificity of 98.92%, a positive predictive value of 93.47%, and an accuracy of 98.87%; the congestive heart failure class was a sensitivity of 99.30%, a specificity of 99.79%, a positive predictive value of 99.19%, and an accuracy of 99.69%.

Yadav et al. [80] implemented a convolutional neural network (CNN) made of two layers of convolution-pooling, two dense layers, and one output layer for diagnosing myocardial infarction using ECG. This network uses Leaky ReLU neurons with categorical cross-entropy loss function and the ADAM optimizer algorithm for better performance. To avoid the problem of overfitting, the L2 method for regularization of the dense layer of CNN was applied. The performance obtained of sensitivity, specificity, and accuracy of 100%, 99.65%, and 99.82%, respectively, for data taken from the training set, and 99.88%, 99.65%, and 99.82%, respectively, on the testing set.

The authors of [84] proposed a set of end-to-end automatic diagnosis algorithms for ECG diseases based on intelligent simulation modeling. The database used in this study was the Center for Communicable Disease Dynamics (CCDD) database. This database contained 193690 12-Lead ECG data with the sampling rate at 500 Hz. This database used two datasets: the first dataset contained 31497 data on disease types; the second dataset contained 43510 data. The features extracted from the ECG data were: QRS width, mean QRS width, RR interval, average RR interval, PR interval, PP interval, TP segment, average TP segment, QRS main wave, R wave time limit, S wave time limit, Q wave time limit, R peak time limit, Q peak voltage value, R peak voltage value, S peak voltage value, QRS main wave voltage, QRS main wave average voltage, Q wave type, R wave type, S wave type, T wave type, P wave type, QRS axis deviation, and ST-segment morphology. For the first dataset, the proposed method has an accuracy of 90.47%, a sensitivity of 81.59, and a specificity of 91.75%. For the second dataset, an accuracy of 89.50%, a sensitivity of 81.18%, and a specificity of 90.71%.

In [91], the authors proposed cardiovascular disease (CVD) detection by using a Deep Neural Network (DNN) with the help of Heart Rate Variability (HRV). The data utilized in this study was collected from 3 PhysioNet databases: Arrhythmia Database from MIT-BIH, Normal Sinus Rhythm Database MIT-BIH, and Congestive Heart Failure Database from BIDMC. With these databases, the author detected the R peaks wavelength for the ECG signal. The experimental results of the Deep Neural Network method achieved 99% accuracy.

In [93], a hybrid neural network approach was proposed by combining two non-specific surrogate Coronary artery disease (CAD) markers, i.e., anomalous ECG morphology and abnormal HRV, in a single CNN-LSTM architecture. This approach was evaluated on two datasets, a corpus selected from the MIMIC II waveform dataset and a partially noisy in-house dataset, recorded using a low-cost ECG sensor. Results showed that overall classification accuracy of 93% and 88% were achieved on the two datasets, which outperformed the existing approaches from the revised literature.

Bouny et al. [98] presented an End-to-End Learning method for heart disease diagnosis from a single-channel ECG signal. The authors proposed a Multi-Level Wavelet Convolutional Neural Network (ML-WCNN) to recognize various cardiac arrhythmias automatically. The ECG database was constructed from MITDB by randomly selecting 45000 ECG fragments distributed as follows: 13200 normal, 7100 premature ventricular contractions, 7200 of ht bundle branch block, 8000 left bundle branch block, 2500 premature atrial contraction, and 7000 paced beats. The Multi-Level Wavelet Convolutional Neural Networks model extracted the features. The methods used were: the convolutional neural networks (CNN), the long-short term memory (LSTM), the deep unidirectional LSTM, the denoising auto-encoder, the deep neural networks, and the wavelet sequence, the convolutional auto-encoder, the faster regions with CNN, and the short-time Fourier transform. The proposed method has an accuracy of 99.57%.

In [104], an artificial intelligence approach for the detection of left atrial enlargement (LAE) based on 12-lead electrocardiography (ECG) was proposed. The dataset from 3,391 older adults over 65 years old who had both 10-s 12 lead ECG and echocardiography was used in this study. The left atrial (LA) anteroposterior diameter >40 mm on echocardiography was diagnosed as LAE, and the LA anteroposterior diameter was indexed by body surface area (BSA) to classify LAE into different degrees. A convolutional neural network (CNN) was trained and validated to detect LAE from normal ECGs. The model's performance was evaluated by calculating the area under the curve (AUC), accuracy, sensitivity, specificity, and F1 score. The proposed method for ECG-identified LAE obtained an AUC of 0.949 (95% CI: 0.911–0.987). The sensitivity, specificity, accuracy, precision, and F1 score were 84.0%, 92.0%, 88.0%, 91.3%, and 0.875, respectively.

Li et al. [107] proposed designing a simple architecture of the deep neural network, CraftNet, for accurately recognizing the handcraft features. This study used the MIT-BIH database available on Physionet. It contained 48 30 min-long 2-lead ECG records samples from 47 patients at 360 Hz. From the ECG signals, the author extracted R-R intervals (RR), wavelet, higher-order statistics (HOS), and morphological (Morph) features. The proposed method could characterize the normal beat, supraventricular ectopic beat, ventricular ectopic beat, fusion beat, average value, and accuracy. The regular beat has positive productivity of 99.18%, a sensitivity of 88.16%, and a specificity of 94.34%. The supraventricular ectopic beat has positive productivity of 41.64%, a sensitivity of 85.37%, and a specificity of 94.85%. The ventricular ectopic has positive productivity of 95.63%, a sensitivity of 94.53%, and a specificity of 99.70%. The fusion beat has positive productivity of 10.89%, a sensitivity of 89.92%, and a specificity of 94.28%. The average value has positive productivity of 61.84%, a sensitivity of 89.25%, and a specificity of 95.79%. The accuracy of this method was 89.24%.

In [109], the authors propose a deep learning algorithm for the classification of heartbeats, combining a convolutional neural network with bidirectional long short-term memory. For this study, the authors utilized two different datasets, one collected from the MIT-BIH arrhythmia database and containing 1000 10-s single-lead ECG segments, and the other shared by the China physiological Signal Challenge 2018 (Liu et al., 2018), including 6877 12-Lead ECG recordings, of which 53.7% were acquired from male participants, and 46.3% were obtained from female participants. The CNN blocks automatically accomplished the feature extraction for these signals. The proposed method achieved, for the first database, a sensitivity, specificity, and F1-score of 84%, 99%, and 85%, respectively, and for the second database, a sensitivity, specificity, and F1-score of 74.3%, 97.5%, and 80%, respectively.

The authors of [117] proposed a coronary artery disease (CAD) and congestive heart failure (CHF) classification method based on ECG fragment alignment (EFA)-principal component analysis (PCA) convolutional network (EFAP-Net). This study obtained data from 3 databases: MIT-BIH Normal sinus rhythm, St Petersburg INCART 12-lead Arrhythmia, and BIDMC Congestive heart failure. The first database has a normal ECG type with 18 2-lead ECG records. The second has coronary artery disease ECG type with 17 2-lead ECG records. The third database has congestive heart failure ECG type with 15 2-lead ECG records. The EFAP-Net method used in this study was an accuracy of 99.78%.

Yao et al. [118] investigated associations of Coronary artery disease (CAD) with increased QT interval variability and waveform ST–T segment abnormalities, such as T-wave inversion and ST-segment elevation or depression, and their efficacy in automated CAD detection. The dataset containing related clinical characteristics and 5-min single-lead ECGs of 107 healthy controls and 93 CAD patients was constructed. Based on this dataset, simultaneous analyses were conducted in five scenarios. Different ML algorithms were applied to classify the two groups with various features derived from the RR and QT interval time series and ST–T segment waveforms. Compared with features obtained from the RR interval time series, better classification results were achieved utilizing those obtained from the QT interval time series. The classification results were elevated by combining the utilization of features derived from the RR and QT interval time series. Further fusing features extracted from ST–T segment waveforms achieved the best performance with 96.16% accuracy, 95.75% sensitivity, and 96.40% specificity. Based on the best performance, an automated CAD detection system was developed with extreme gradient boosting, an ensemble ML algorithm, and the residual neural network, namely, a deep learning method.

Deb et al. [123] developed a method to classify ECG signals into two groups: normal and abnormal. Angina, Bundle Branch Block, Cardiomyopathy, Heart Failure, Dysrhythmia, Myocardial Hypertrophy, Myocardial Infarction, Myocarditis, and Valvular Heart Disease. All these cardiac conditions were classified as abnormal ECG signals. Statistic features skewness, kurtosis, the standard deviation of detail, and approximation coefficients of the Daubechies wavelet (db10) of order 5 for several abnormal and normal ECG signals were obtained in the extraction stage. Support Vector Machine (SVM) was used for model classification. This method's accuracy, sensitivity, and specificity were compared and evaluated by testing the SVM with 36 signals obtained from the MIT-BIH Normal Sinus Rhythm Database and 36 signals from the PTB Diagnostic ECG Database, which yielded an accuracy, sensitivity, and specificity of 98.61%, 97.37%, 97.22%, respectively.

In [125], the authors proposed models of combinations of multi-lead ECG from the 12-lead ECG St. Petersburg Arrhythmias database to detect premature ventricular contractions (PVCs) and optimize the required data pre-processing resources for Convolutional Neural Network(CNN) implemented on wearable devices. Was considered two scenarios to evaluate the performance of data processing for CNN classification of PVCs. Firstly, Wavelet Fusion was applied for 6-lead ECG to be our CNN input image, and second, was used Tensor decomposition of multi-leads combination techniques for the same 6-lead ECG. CNN model was built with TensorFlow to classify our ECG signals with or without PVC. The designed CNN has 7 layers, 2 convolutional layers, 2 max-pooling layers, 1 fully connected, and 1 output layer. CNN's architecture and setup parameters were the same for all the cases: Factors as input and Fused Wavelet Image as input. 5-fold cross-validation was used for tuning the hyperparameters of CNN. Experimental results showed that tensor decomposition of stacked multi-lead ECG achieved better performances than wavelet fusion for 6-lead ECG. The achieved accuracy for the Tensor-based method reaches 90.84% with a sensitivity of 78.6% and a specificity of 99.86% using the SELU activation function.

The authors of [129] proposed a deep convolutional neural network-Recurrent neural network (CNN-RNN) model for automatically staging heart failure diseases in real-time and dynamically. The dataset utilized in this study was from the chest pain centers (CPCs) of the Shanxi Academy of Medical Sciences. The data collected included 573 patients at least 18 years old from January 2013 to December 2017, including healthy persons and heart failure patients. The features were extracted automatically using the automatic CNN-RNN method. This method has an accuracy of 97.6%, a sensitivity of 96.3%, a specificity of 97.4%, and a positive predictivity of 97.1%.

In [133], the authors present a method for cardiovascular disease classification, proposing an end-to-end trainable cross-domain transfer learning for cardiovascular diseases from ECG waveforms by utilizing existing vision-based Convolutional Neural Network Frameworks as feature extractors, followed by ECG feature learning layers. The authors collected ECG signal data from two separate datasets, the ICBEB and CCH datasets. The ICBEB dataset contains 12-Lead ECG records from a total of 6877 subjects, of which 5959 were diagnosed with at least one of the following cardiovascular abnormalities: Atrial fibrillation, First-degree Atrioventricular Block, Left Bundle Branch Block, Right Bundle Branch Block, Premature Atrial Contraction, Premature Ventricular Contraction, ST-segment Depression and ST-segment Elevated. The GGH dataset has ECG signals collected from a total of 21241 patients, and for this study, the data from 11,853 Myocardial Infarction and 5,528 normal patients was used. The Softmax layer automatically extracted the relevant ECG features from these signals. The proposed method is then validated on both datasets, achieving an accuracy of 49.9% for the ICBEB dataset and 85.8% for the GGH dataset.

Tison et al. [134] developed and tested an algorithmic framework named ecgAI that facilitates scalable analysis of ECG data while preserving interpretable parallels to cardiac physiology. The data was obtained from The University of California, San Francisco (UCSF) ECG database and was selected for 36186 12-Lead ECG. The authors developed a 725-component ECG vector representation derived from the CNN/HMM segmented ECG segments to extract features: the PR interval, the QRS complex, the ST-T wave complex, and the TP segment. The results of the proposed method, ecgAI, were 91 ± 3 for the P wave, 85 ± 2 for the PR segment, 94 ± 4 for the QRS complex, 88 ± 3 for the ST segment, 91 ± 3 for the T wave, and 92 ± 5 for the TP segment.

In [136], the authors proposed an ECG-based classifier for Congestive Heart Failure, utilizing Stockwell Transform and Hybrid Classification Scheme. For the study, congestive heart failure and normal sinus rhythm ECG signals were collected froth the Beth Israel Deaconess Medical Center (BIDMC) CHF database and the Massachusetts Institute of Technology-Beth Israel Hospital (MIT-BIH) arrhythmia database, respectively. The BIDMC CHF database comprises two-lead ECG signals collected from 15 subjects lasting an hour and 6 min each. The MIT-BIH arrhythmia database contains 17 NSR ECG signals, each lasting 30 min. To utilize the Hybrid classification scheme, the authors extracted the time-frequency entropy features from these signals, with the 48-dimensional feature vector, with the entropy features computed from the first 48 frequency components, serving as the input for the hybrid classifier. The proposed method achieved an accuracy of 98.78%, a sensitivity of 98.48%, and a specificity of 99.09%.

The authors of [141] proposed a deep learning method to create an automated system for detecting and classifying cardiovascular diseases. The CNN made to detect cardiovascular diseases was trained to utilize 259,789 ECG signals from a dataset constructed by the authors from the ECG management system of the First Affiliated Hospital of Nanjing Medical University, with a total of 277,807 12-lead static ECG recordings. Having applied the proposed method, the authors reported an accuracy of 98.27%, a precision of 60.93%, and a sensitivity of 99.95%.

Hao et al. [147] propose a method for classifying cardiovascular diseases called the Softmax regression model, which utilizes the available state data of the two-layer neural network structure of the Softmax regression model for training and learning, and to then calculate the probability of reclassification data belonging to each category. The categories mentioned correspond to the maximum likelihood and the classification result of the data to be classified. The ECG signal data from the MIT-BIH database is utilized during this study, with the authors extracting ECG data of patients with 3 kinds of cardiovascular diseases: atrial fibrillation, normal sinus rhythm, and ventricular two rates. The feature extraction from this data is then automatically carried out by the Softmax regression model. Having applied the proposed method to the database, the authors report a correct classification rate of 94.44%.

The authors of [149] propose a heart disease classification method using Long Short-Term Memory. The ECG signals utilized during this study were extracted from the UCR time series classification archive, from which the authors randomly selected 5000 heartbeats. The ECG features were then extracted from these signals by an automatic classification process called symbolic aggregate approximation. The accuracy achieved by this method was 97%.

Acharya et al. [158] proposed an automated ECG classification method for coronary artery disease by applying Higher-Order Statistics and Spectra. For this study, the authors utilized the ECG signal data from the St. Petersburg Institute of Cardiological Technics 12-lead Arrhythmia Database to extract 17 coronary artery disease records from 7 subjects. The Fantasia, an open-access database, extracts 40 normal records from 40 subjects. From these ECG signals, the authors extracted a total of 136 bispectra and 136 cumulant features. The proposed method was able to characterize the normal and coronary artery disease-affected ECG signals with a 98.17% accuracy, a 94.57% sensitivity, and a 99.34% specificity using 13 bispectrum features, and an accuracy of 98.99%, a sensitivity of 97.75%, and a specificity of 99.39% using 31 cumulant features.

In [161], the authors proposed a methodology for automating the diagnosis of normal and Coronary Artery Disease conditions using Heart Rate Variability signals extracted from ECGs. The authors obtained the coronary artery disease beats from the Long-Term ST Database, consisting of 86 recordings from 80 human subjects, of which 46 were men, ages 44–85, and 29 were women, ages 23–87. To create a coronary artery disease group, only 23 of the 80 subjects were of interest. The data for the control group of normal beats was acquired from 24-h Holter monitor recordings of 54 healthy subjects, 30 of which were male and 24 of which were female, ages 29–76, with a mean age of 61. To create groups of equal size, the authors only extracted 23 beats from this database. The authors then extracted different features from these signals that could be organized into three groups: time-domain, frequency-domain, and nonlinear characteristics. The time-domain features extracted the standard deviation of normal to normal R-R intervals, the standard deviation of successive RR interval differences, the square root of the mean of the sum of the squares of differences between adjacent NN intervals, the square root of the mean of the sum of the squares of differences between adjacent NN intervals, the baseline width of the RR histogram evaluated through triangular interpolation and the number of all NN intervals/maximum number; the frequency domain features extracted the total power, high frequency, and low-frequency value and ratio of low-frequency power to high-frequency power; and the nonlinear features extracted were the point care plots, recurrence quantification analysis, approximate entropy, sample entropy, detrended fluctuation analysis, and correlation dimension. The authors utilize principal component analysis to reduce the dimension of the extracted features and then, applying a support vector machine, classify the beats into two classes. This method achieved an accuracy of 99.2%, a sensitivity of 98.43%, and a specificity of 100%.

The authors of [163] proposed a method for cardiac disease classification based on ECG signals, allowing for the distinction between 17 classes, and utilizing evolutionary-neural systems. The MIT-BIH Arrhythmia database was used during this study, consisting of records from 45 patients. For feature extraction, the authors used the following methods: Power spectral density, Welch's method, Periodogram, Fourier discrete transform, Hamming window, and Series of logarithms of signals. The best evolutionary-neural system, based on the SVM classifier, obtained a recognition sensitivity of 17 myocardium dysfunctions at a level of 90.20% (98 errors per 1000 classifications, accuracy of 98.85%, specificity of 99.39%, and time for classification of one sample = 0.0023 [s]).

In [169], the authors proposed a method for automatic diagnosis of coronary artery disease, implementing a long short-term memory with a convolutional neural network for that purpose. The authors extracted the normal ECG data used in this study from the Fantasia open-source database and the coronary artery disease ECG data from the St Petersburg Institute of Cardiology Technics 12-leads arrhythmia database. The authors only utilized 7 coronary artery disease records from the database, 6 of which had been obtained from female subjects, 1 which had been obtained from a male subject, and 40 normal beat records, which had been collected from 20 female and 20 male subjects. The relevant features for this study are automatically extracted from these ECG signals by the convolutional layer of the CNN. The proposed method can detect coronary artery disease with a precision of 99.85%, a recall of 99.85%, an F1-score of 99.52%, and an accuracy of 99.85%.

## 4 Discussion

### 4.1 Interpretation of the results

ECG data can be used for different kinds of research, where the monitoring of diseases is revealed as possible. The mainly used sensors combined with ML/DP methods vary between 1- and 12-lead sensors. As the sensors have low costs, they can be used for research purposes, allowing the creation of different solutions.

**Fig. 2:**
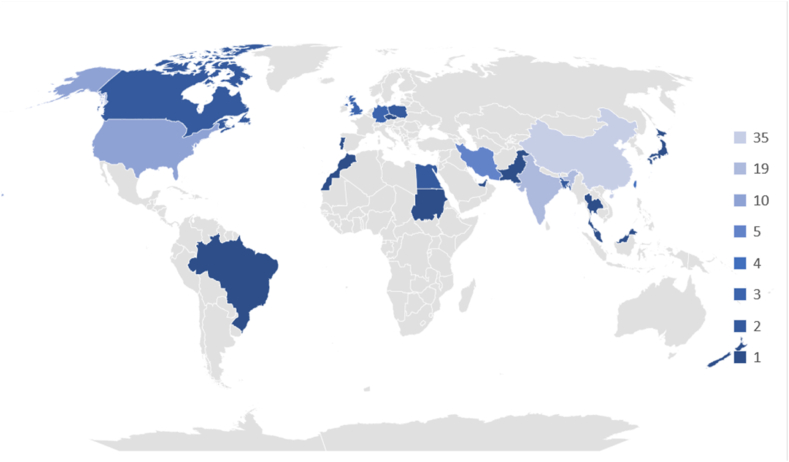
Distribution of studies by the country where the studies were conducted.

Among the 103 studies analyzed, as presented in Fig. 2, thirty-five studies were performed in China, nineteen studies took place in India, ten studies were conducted in the United States of America, five studies were conducted in Iran, four studies were performed in Taiwan, three studies were conducted in Germany, three studies were conducted in Singapore, three studies were conducted in the United Kingdom, two studies were performed in Bangladesh, two studies were performed in Canada, two studies were performed in Egypt, two studies were performed in Poland, and one study for each country was conducted Brazil, Czech Republic, Hawaii, Japan, Korea, Malaysia, Morocco, New Zealand, Pakistan, Portugal, Sudan, Thailand, and United Arab Emirates.

**Fig. 3:**
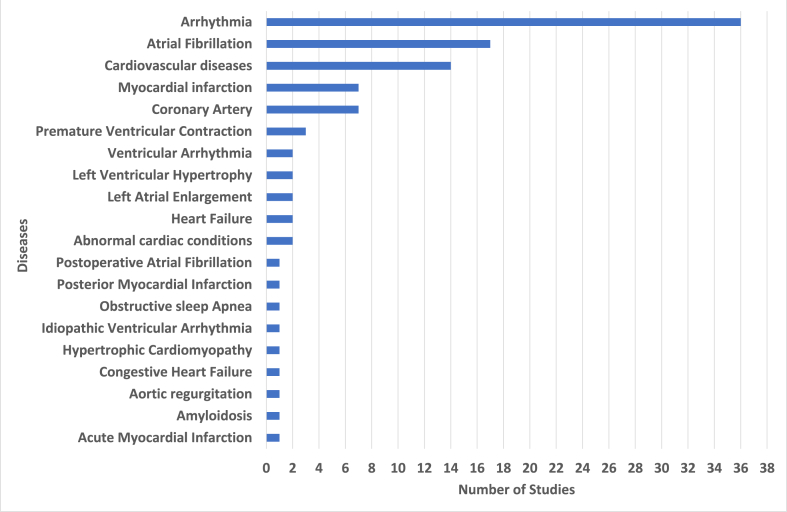
Distribution of studies by disease.

Some studies were based on problems related to a specific disease, and Fig. 3 demonstrates various diseases that were conducted in the studies, where thirty-six studies analyzed arrhythmia, seventeen studies examined atrial fibrillation, fourteen studies analyzed undefined cardiovascular diseases, seven studies analyzed myocardial infarction, seven studies analyzed coronary artery, two studies analyzed ventricular arrhythmia, two studies analyzed left ventricular hypertrophy, two studies analyzed left atrial enlargement, two studies analyzed heart failures, and two studies analyzed abnormal cardiac conditions. The remaining diseases were diagnosed in one study.

Regarding Table 3, most studies relied on datasets/databases to perform the tests. It is essential to mention that MIT-BIH databases had the most usage, counting forty studies. MIT-BIH arrhythmia is, by far, the most accessed database. Thirteen studies did not use any database, relying only on the population.

**Table 3:** Relations between datasets and studies.

Datasets	Studies
MIT-BIH arrhythmia database [48,56,88,97]	[23], [47], [49], [50], [65], [74], [82], [86], [89], [91], [95], [98], [99], [103], [107], [108], [109], [112], [113], [114], [115], [122], [124], [131], [137], [142], [151], [152], [157], [163], [164]
PhysioNet database [41,56,61,81,110,128]	[40,60,77,121,125,127,132,153,159]
Physikalisch-Technische Bundesanstalt (PTB) database [28,29,55,56,81]	[39,54,58,80,87,111,116,162]
MIT-BIH Atrial Fibrillation database [56,73]	[72,95,100,101,147,156]
Medical Center (BIDMC) CHF [63]	[60,91,117,136]
ICBEB [24]	[109,119,133,138]
Fantasia database [69]	[68,158,169]
MIT-BIH Normal Sinus Rhythm database [62]	[60,91,117,123]
St. Petersburg Institute of Cardiological Technics [70]	[68,158,169]
CPSC 2018 dataset [83]	[82,88,90]
Creighton university ventricular tachyarrhythmia database [75]	[74,78,95]
MIMIC II waveform dataset [94]	[93,120]
Atrial Fibrillation Prediction database [144]	[143]
CCDD [85]	[84]
CHIEF Heart Study [45,46]	[44]
ECG-ViEW II database [106]	[105]
Guvenir et al. dataset [146]	[145]
Hefei Hi-tech competition dataset [67]	[66]
San Francisco database [135]	[134]
UCI machine learning repository [167]	[166]
UCR Time Series Archive [150]	[149]

**Fig. 4:**
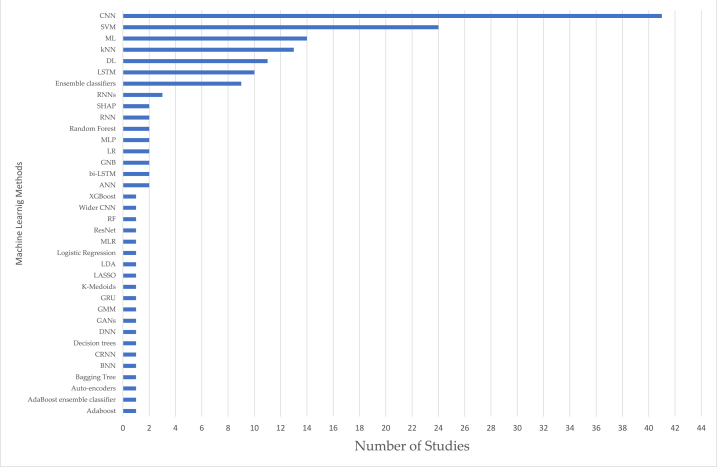
Distribution of the various machine learning methods by the studies.

Another noteworthy mention is the methods used for the interpretation of these studies. They all share the same method but use different implementations. Machine learning showed an excellent correlation when working with ECG data. Concerning Fig. 4, the studies used different implementations, being the convolutional neural network (CNN) the most common madding appearance in forty-one studies, followed by support vector machine (SVM) appearing in twenty-four, and classical machine learning (ML) appearing in fourteen studies as well.

### 4.2 Comparison of the different studies

In Table 4, we summarize the main results and limitations of the selected relevant studies related to the disease identification, classification, and recognition of different diseases with ECG data.

**Table 4:** Discussion of study results and limitations.

Paper	Results and Benefits	Limitations
Anand et al. [39]	The proposed model produced better results than the other state-of-the-art models on the same datasets. This model demonstrated generalizability by achieving good results on two different ECG dataset. The proposed model can be easily integrated with the existing ECG machines, to help the doctors in primary and secondary healthcare.	N/D
Geweid et al. [40]	The results showed that the proposed method was the high reliability and accuracy which simplifies the extraction process and removal of detecting ECG signal fiducial points and removing hand-crafted features. This algorithm could be applied to personal health monitoring systems as it had reliably detected atrial fibrillation as well as other rhythms on ECG recording.	N/D
Guo et al.	The proposed model showed a clearly useful discriminative performance (C-statistic >0.75) in the training [C-statistic 0.857 (0.818–0.896)], and temporal validation cohorts [C-statistic 0.871 (0.812–0.930)]. In the external validation cohort, the C-statistic of the model was 0.833 [0.825–0.841].	Further external validation using participants from multiple centers and a population with more heterogeneity is needed. The analysis of ECG variables in the current study focused only on those parameters that are easily assessed in clinical practice; thus, some important but less frequently used variables might have been omitted. The proposed model showed high sensitivity for HCM screening, at the cost of a high false-positive rate
He et al. [43]	The results of this study showed that long-term ECG monitoring could significantly improve the detection rate of postoperative atrial fibrillation. The model combining P wave parameters and clinical data performed better in predicting postoperative atrial fibrillation.	The included sample size was relatively small, which may affect the results. ECG signal quality limited the effective of machine learning model.
Hsu et al. [44]	This was the first study using machine learning for ECG and biological features to predict Left Atrial Enlargement (LAE) early in young adults. The SVM was the best machine learning classifier for ECG features only to detect LAE in young males, achieving an AUC of 78% of the ROC. In contrast, the MLP was the best machine learning classifier, which could improve the performance from 73 to 81% after biological features were added to the MLP model.	Population of military males were physically active, the living environment was a closed system, the participants had a similar daily schedule, and the unmeasured bias could be minimized. As data were only obtained from the males, the results might not be the same for the females. Oxidative stress was also related to the occurrence of atrial fibrillation, and this was not considered in this study.
Li et al. [47]	The proposed method was illustrated by high classification performance under inter-patient paradigms, even though single-lead raw ECG data was used only. The advantage of this method was that it could classify arrhythmias without extracting heartbeats, and the classification accuracy was comparable to that of the methods mentioned in the introduction.	The method proposed in this paper needs to use a larger annotated heartbeat database to improve the classification performance.
Liu et al. [49]	Using the comparative experiments, the proposed method achieved the optimal effect in the investigation of the same type of methods. This method had higher accuracy than other methods, and it was simpler and easier to understand.	The proposed method does not use deep learning methods to classify arrhythmia heartbeat.
Mazidi et al. [50]	The experimental results reveal that the combination of the proposed method with the KNN classifier had better performance than other methods.	A limitation was that the proposed method had limited performance with the processing tools in real time due to the complexity of mathematical computations in the TQWT algorithm.
Sawano et al. [51]	The area under the receiver operating characteristic curve of the multi-input model was significantly greater than that of the proposed model alone and those of other machine learning models. This study may be a first step toward creating a screening tool for aortic regurgitation, facilitating early diagnosis.	First, because this study was a single-center retrospective, there may have been patient selection bias. Second, the authors had no data regarding the patient's heart failure status. And the last limitation was the Grad-CAM method showed that the multi-input model focused on the QRS complex in leads I and aVL and the analysis of single-lead models and variable importance showed consistent findings.
Zhao et al. [52]	The proposed CNN- LSTM model predicted left ventricular hypertrophy (LVH) with higher sensitivity than the Cornell voltage criteria and Sokolow-Lyon voltage (68, 48, and 14%, respectively), whereas its specificity was inferior to these two criteria (57, 72, and 96%, respectively).	This was a single-center study, the models may have the risk of generalizing poorly to other hospital systems and other datasets The accuracy of proposed model still needed to be improved.
Zheng et al. [53]	In comparison of the location prediction performance between human experts and proposed machine learning algorithm, results showed that the sensitivity, specificity, F1-Score, and accuracy of the machine learning-enabled ECG approach exceeded those of the human experts 0.57, 18.18, 2.17, and 3.95%, respectively.	A multi-center prospective evaluation in larger cohorts is necessary to show robustness and compatibility of the proposed algorithm.
Dey et al. [54]	The proposed model provides relevant information of temporal features, and their correlation with the physical state of the myocardial infarction patients could provide a great help for the specialists.	This method shows promising results if a selection of a minimum of 12 consecutive ECG cycles can be ensured for proper extraction of all the features used.
Che et al. [57]	The proposed model combines CNN and Transformer networks to extract temporal information in ECG signal and can perform arrhythmia classification with acceptable accuracy. This model can help cardiologists perform diagnosis of heart disease, improving the efficiency of healthcare delivery, and the authors hope it can be applied to low-cost ECG devices.	N/D
Chen et al. [22]	The results showed that the proposed method performance was better for both classifications of ECG signals. This study brings a greater burden to the evaluation of doctors and the management of medical institutions.	N/D
Dai et al. [58]	The proposed end-to-end model enables the fast prediction of diseases without manual feature extraction. The results reported high accuracy, sensitivity, and specificity.	N/D
Grogan et al. [59]	The results demonstrated the ability of an AI-ECG tool to detect amyloid heart disease before clinical diagnosis. The use of this model to detect cardiac amyloidosis may promote early diagnosis and initiation of potentially lifesaving therapy.	The number of ECGs was limited. It was possible that individuals with unrecognized cardiac amyloidosis may have been included in the control group. Some patients with amyloid in the training set may not have had definite cardiac involvement. Fourth, the model needed validation in larger cohorts with prospective application.
Haleem et al. [60]	The achieved results help to reduce domain experts' work, by computing useful signal characteristics via an automated complete system for early diagnosis of CVD. This tool had the capability to be implemented under real-time settings and to be tested in patients with known heart disease.	The publicly available datasets used in this study presented some missing information and limitations.
Houssein et al. [65]	The experimental results revealed that the proposed method achieved good results compared with the competitor algorithms. And demonstrated a promising use for professionals who want to diagnose heart diseases based on the ECG signal.	N/D
Hua et al. [66]	The proposed a feature selection framework can extract specific clinical metric features from raw ECG data, these features are more concrete and has their meaning compared to the ones extracted by other deep neural networks.	The number of the features extracted from the raw ECG data give a small subset of what the samples can offer.
Jahmunah et al. [68]	The proposed method was more effective than the CNN model, as it can be trained faster with lesser weights and achieving high accuracy performance. This method was preferred for the classification and can be potentially used as an assistive tool for clinical experts to confirm their diagnostic decisions quickly.	The first limitation, the authors just used a few subjects for CAD and CHF groups. The second limitation, the necessity of having a large dataset to train and test the proposed method.
Li et al. [71]	The proposed algorithm BiLSTM–CNN can diagnose multiple types of cardiac arrhythmias with promising accuracy of 99.56%, clinical value, and robustness, which may be potentially useful in assisting risk stratification, clinical diagnosis, and real-time ECG monitoring.	The proposed model still needs to be further tested and improved by using other ECG datasets with more types of rhythmic abnormalities.
Luo et al. [72]	The proposed model outperformed some state- of-the-art studies, with a high overall performance value. The results achieved demonstrated that the SMOTE technique can improve the classification accuracy of the method, especially for the minority classes. This method is an effective tool for performing rapid and consistent arrhythmia diagnosis that can help cardiologists correctly identify heartbeat types.	This method requires a large amount of data and the time cost of the training phase and the model's training by using 10-fold cross validation is high and demands powerful computers
Naz et al. [74]	The results of the proposed method had a higher accuracy than existing methods, and the execution time is minimized.	N/D
Nguyen et al. [76]	The training strategy applied to the convolutional neural network during this study resulted in the extraction of useful deep features without the need for expertise on ECG signals and cardiac rhythm disorders. Stacking SVN with RBF kernel on the statistics of CNN predictions allowed for precision in distinguishing different heartbeat classes. When compared to other methods submitted for the Physionet 2017 challenge, this method achieved better scores than most. The proposed method could be extended to other problems related to medical signals.	The authors reported a weakness with the method, although they say it was due to the nature of the dataset. The lack of information related to the location of the atrial fibrillation rhythm, as well as other rhythms in each ECG, may have resulted in some segments from atrial fibrillation signals not containing atrial fibrillation. Similarly, a segment from an others signal may not contain any other rhythms.
Radhakrishnan et al. [77]	The proposed method demonstrated higher classification performance using chirplet transform-based time-frequency representation of ECG signal as compared to other methods. The proposed method could be deployed in intelligent healthcare systems for automated atrial fibrillation detection using ECG sensor data.	N/D
Sabut et al. [78]	The results reached an accuracy of 99.2%, which was better than other studies. The proposed method could be improved further in terms of detection accuracy and computational complexity.	N/D
Yadav et al. [80]	Sensitivity, specificity, and accuracy of 99.88%, 99.65%, and 99.82%, respectively, on patients, it hasn't seen before, which suggests that the model can achieve excellent classification performance.	During the handling of ECG signals, noise reduction was an important issue.
Wang et al. [82]	The proposed method's experimental results demonstrated that it achieved better performance values than the two control groups and various state-of-the-art algorithms. This study introduced the scale parameter into the Bidirectional Gated Recurrent Unit Neural Network model that allows it to achieve a better trade-off between model performance and computation cost than Gated Recurrent Unit and Bidirectional Gated Recurrent Unit Neural Network.	The proposed model only detects Premature Ventricular Contractions and its generalization ability could be improved utilizing more diverse and abundant data sources.
Wang et al. [84]	The proposed method had a lower computational load and higher interpretability. It wasn't affected by data imbalance and small sample size and had real-time performance.	The proposed method accuracy was affected by localization.
Wu et al. [86]	The proposed convolutional neural network was able to successfully process the non-filtered dataset, and the ten-fold cross-validation implemented in this work proves the robustness of the network. This study benefits the research community by reporting results on the classification of micro-classes of Arrhythmia that were normally ignored.	A limitation of the proposed model arises from the computational costs of training such a network, due to large quantities of data.
Xiong et al. [87]	The proposed method achieved superior results than other methods, with an accuracy of 99.87%. This method could be introduced into clinical practice to assist the diagnosis of myocardial infarction.	N/D
Zhang et al. [88]	The results showed that the proposed method enables the model to automatically extract various types of inter-layer complementary multi-scale features from ECG signals in a global space and facilitates the improvement of the final classification performance. The proposed model had tremendous potential to be applied to ECG analysis platforms in hospitals to achieve more accurate and robust atrial fibrillation detection.	The training data was restricted to single-lead ECG recordings, which provides limited spatial information compared to a standard 12-lead ECG. Due to the lack of sufficient data from other categories it was only possible to focus on the detection of atrial fibrillation abnormalities.
Zhang et al. [89]	The proposed method achieved an accuracy of 99.8% for ventricular ectopic beat detection, and an accuracy of 99.7% for supraventricular ectopic beat detection.	N/D
Zhang et al. [90]	The results showed that the proposed method had a better performance than other classifiers.	Since the dataset was entirely collected from China hospitals, the study didn't have data for different races. And adversarial samples could lead to misbehaviors of deep learning models.
Ambhore et al. [91]	The proposed method achieved better results when attempting to detect cardiovascular diseases than other famous machine learning techniques. The pre-processing of the ECG peaks as an entry vector to the DNN allowed for the identification of morphological characteristics useful in forecasting attitudinal stress. The experimental results validated the proposed methods and resulted in an improvement in CVD classification for the MIT- BIH Dataset.	N/D
Banerjee et al. [92]	CNN and Long Short-Term Memory methods increased the precision and accuracy of a lightweight wearable Arrhythmia Detector model that classifies real-time ECG signals and the mobile application made it more convenient.	The proposed model can be upgraded with better components for recording ECG signals which further increases accuracy due to its low-noise and accurate output.
Banerjee et al. [93]	Results showed that overall classification accuracy of 93% and 88% are achieved on the two datasets, which outperform the existing approaches.	The biomarkers considered in this work are not guaranteed at the onset of CAD and the proposed approach failed to detect few of the borderline patients.
Bitarafan et al. [95]	The performance of the proposed model on test samples is 98.93%, 99.78%, and 99.58% respectively in terms of overall accuracy, sensitivity, and specificity for tackling the problem of 4- class arrhythmia classification.	N/D
Deng et al. [100]	The results show that the sensitivity, specificity, and total accuracy of the proposed method were 99.07%, 97.05% and 98.03%, respectively.	N/D
Bouny et al. [98]	The results showed that the proposed method could classify six types of ECG beats with higher recognition accuracy of 99.67%.	The proposed methods required long training time, which was computationally expensive. The validation technique required a long time to evaluate the performance of the systems. The visualization technique required an expensive time to be processed for a large testing set.
Chumrit et al. [99]	The best detection results based on the average energy feature with 10-fold cross validation presents are 96.67% average accuracy, 93.33% sensitivity, 100% specificity and 100% precision.	N/D
Hatamian et al. [102]	The result showed that oversampling, GMM and DCGAN augmentation algorithms on ECG signal classification into AF and Normal classes improve the performance.	In some cases, using GAN and GMM to augment the AF class causes slight deterioration of the Normal class accuracy.
Hammad et al. [101]	The average accuracy values of 98% showed the efficiency of proposed technique for arrhythmia detection from the MIT-BIH dataset. Performance of the model was reported in terms of specificity (98.9%) sensitivity (99.7%) and positive predictivity (95.8%) for the five-fold cross-validation.	N/D
Hsu et al. [103]	The proposed method achieved an accuracy of 98.8% and a sensitivity of 96.3% for class V, and a sensitivity of 98.6% for class Q. The authors also found the key waveform parameters that contribute to arrhythmia classification with the proposed WBSP method.	N/D
Jiang et al. [104]	The results showed that AI-enabled ECG performed well in diagnosing left Atrial Enlargement (LAE) achieved 95% of accuracy, especially in diagnosing moderate and severe LAE.	The proposed model requires further refinement and external validation.
Ibrahim et al. [105]	The CNN model showed competitive F1 score of >89%, sensitivity >88%, and specificity >93% beating literature approaches. The best performance showed XGBoost model with an F1 score of 97.1%, sensitivity of 93.5%, and specificity of 99.4%.	The RNN model underperformed when compared to the CNN model, with an F1 score of 89.0%, sensitivity of 93.2%, and specificity of 88.1%. due to its more fitting application to time-series data and not static data.
Li et al. [107]	The results demonstrated that the proposed method effectively faced the data imbalance problem. The results showed that the proposed approach had stronger classification ability than the other methods and was less influenced by the data imbalance used in this study.	N/D
Li et al. [108]	The accuracy and training time before incremental learning of proposed CNNBLS were 97.94% and 21.61 s, and the accuracy and training time after incremental learning with additional 12929 data were 98.45% and 47.23 s, which overperformed traditional deep learning networks in term of time-consuming CNN 696.95 s, LSTM 409.89 s, and an accuracy 98.93%, 97.87% respectively.	Despite CNNBLS showed the better results for accuracy at incremental training sets model, compared with initial data training model, the time-consuming was increased from 21.61s to 47.23 s
Liang et al. [109]	The proposed method was able to process the data quickly, and it achieved good performance with an overall mean F1 score of 80%. The proposed method was a generic method that could be used for other bio signal applications.	Not as accurate as other methods tested, but faster.
Prabhakararao et al. [111]	A notable advantage of proposed method was that it provided robust discrimination between MI and non-MI patients and its model interpretability, which correlated with the clinician's way of inspecting the 12-lead ECG for the diagnosis.	The proposed method has a moderate number of parameters with a slightly higher average run-time of 8.42 ms to provide a classification decision.
Mazaheri et al. [112]	This study achieved better accuracy than other methods. The proposed method could help physicians improve the diagnostic accuracy of the clinical decision-making process.	Not employing the entire MIT-BIH database signals and diagnosis; only seven classes of arrhythmias were one of the main disadvantages of this study. And the proposed method could not detect the ECG signals with more than one class type abnormality.
Rahman et al. [113]	The proposed CNN classification model achieved an overall classification accuracy of 95.2% with an average precision and recall of 95.2% and 95.4% that significantly outperforms the identified in state-of-the-art methods.	N/D
Subramanian et al. [114]	The performance of proposed SVM model was an accuracy of 91% with precision recall and F1 score of about 0.906593.	N/D
Wang et al. [115]	The results demonstrated that the proposed method had high performance for arrhythmia detection. The proposed method could interfere with the classification effect for a certain disease, which had advantages for the classification of rare classes.	Unbalanced datasets.
Wang et al. [116]	The proposed method had better results than other methods, with an accuracy of 99.71%. The combination of the proposed method and hardware devices facilitated the automatic diagnosis of ECG for clinical applications.	The proposed method was too complex.
Yang et al. [117]	The studies showed that the proposed method had a fast heartbeat classification speed, and it had significant noise robustness. This method could be conveniently applied in the clinic or on mobile devices	N/D
Yao et al. [118]	The best classification results were achieved using a combination of above features derived from the RR and QT interval time-series and the ST–T segment waveforms, with 96.16% accuracy, 95.75% sensitivity, and 96.40% specificity.	A dataset with a larger subject population would certainly improve the results. The investigation was conducted on coronary artery disease (CAD) patients without myocardial infarction (MI), in comparison to healthy control subjects; thus, presented findings might not be applicable to MI patients. The findings were only tested on the constructed database.
Zhang et al. [119]	The authors proved that the proposed model STA-CRNN resulted in superior detection performance in comparison with state-of-the-art methods, especially when it came to identifying arrhythmias with lower recognition rates. This is a promising method with the potential to assist diagnosis.	N/D
Bashar et al. [120]	SVM with the RBF kernel has the best outcome, resulting in 99.88% sensitivity, 99.65% specificity and 99.75% accuracy.	N/D
Boppana et al. [121]	According to results, the proposed combined method could be used to reduce the computation complexity and enhance the precision by using the hyper tuning parameters.	Correlation between obstructive sleep apnea and myocardial infarction could be explored.
Celin et al. [122]	The proposed method on RF classifier yielded an improved accuracy of 98.83% on the signals from MIT-BIH arrhythmia database.	N/D
Deb et al. [123]	The performance of the proposed SVM model yielded an accuracy, sensitivity, specificity 98.61%, 97.37%, and 97.22%, respectively.	In comparison with selected state-of-the-art methods by authors, the performance of the proposed method did not show significant overruns.
Gao et al. [124]	The results demonstrated that the LSTM network with FL reached a reliable solution to the problem of imbalanced datasets in ECG beat classification. This method could be used in telemedicine scenarios to assist cardiologists.	The study was only conducted for eight ECG beat types, and the proposed network was the time cost of the training phase.
Hoang et al. [125]	The proposed method achieved accuracy to detect Premature ventricular contractions (PVC) for tensor-based feature extraction was 90.84% with a sensitivity of 78.60% and a specificity of 99.86%.	The overall performances did not compete with related work on neural network for premature ventricular contractions detection reaching accuracy of 98 and 99%.
Kong et al. [126]	The results demonstrated that the predictive performance of the proposed method was comparable to SVM, and the RVM was more suitable for online diagnosis since RVM was sparser than SVM. The proposed method had a better performance than other methods. The research could be applied to clinical diagnosis of atrial fibrillation.	The limitation of the RVM model was the iterative computation to obtain the weights.
Mahmood et al. [127]	Simulation results showed that the AdaBoost classifier provides a better solution to predict problem class in heartbeat classification obtained a mean improvement report for all classes in testing set 97.3% in area under curve accuracy, 94.7% in classifier accuracy, 96.7% in sensitivity, and of 98% in positive predictive value.	The study of adaptive beat size segmentation is still required. The relationship between underlying physiology and features extracted must be explored.
Li et al. [129]	The results showed that the proposed method achieved good robust and generalization performance on real datasets. Feature extraction, selection and classification procedures were combined in a single deep structure. Denoising was not required. Ten-fold cross validation ensured the results were reliable and robust.	The proposed method required a lot of data for training and took more time to train the data.
Nankani et al. [130]	The proposed method produces an F1 score of 0.88 ± 0.02 on PhysioNet Computing in Cardiology Challenge 2017 database, which is better than the existing methods in the revised literature.	A dynamic neural network could also be employed to add or prune the filters during the training phase to obtain a smaller model that can be used in the mobile devices.
Pandey et al. [131]	The proposed method was fully automatic, and it was not required of an additional system like feature extraction, feature selection, and classification. This method had less computational complexity and could be used for classifying long term ECG signals and detecting disease events in real time.	N/D
Prabhakararao et al. [132]	The results showed that the best test accuracy of 96.69%, sensitivity of 80%, and geometric mean of 88.72% are achieved by WSVM classifier with radial basis function (RBF) kernel.	N/D
Tadesse et al. [133]	The results demonstrated that competitive performance was achieved using transfer learning without training a dedicated network from scratch.	N/D
Tison et al. [134]	The objective of this study was to demonstrate how to extract more knowledge from the data, and yet remain transparent to physicians, patients, and researchers on the provenance of this knowledge.	The machine learning used was just optimized to analyze ECGs in normal sinus rhythm. Also, the data was derived from a single medical center.
Tripathy et al. [136]	The features utilized in this study, entropy features in the range from 10 Hz to 30 Hz, were highly affected by congestive heart failure, so, the hybrid classification method utilized by the authors, based on these features was able to achieve its highest performance values. The time-frequency features the authors extracted using the proposed method can be utilized in the detection of other anomalies in ECG signals.	The proposed method does not predict congestive heart failure when the patient has myocardial infarction, cardiomyopathy, and valvular disease.
Wang et al. [137]	The results showed that the proposed method could be employed as a tool to automatically detect different kinds of arrhythmia when properly trained. It was demonstrated that the proposed method was insensitive to noise, and filtering could be applied before the method.	N/D
Wang et al. [138]	The results of proposal method achieved an average F1-score of 81.3% in classification of 8 types of arrhythmias and sinus rhythm.	N/D
Wu et al. [139]	The random forest prediction model that the authors implemented reached higher sensitivity and accuracy values for the detection of left ventricular hypertrophy than other methods previously proposed.	There were many missing values in electrocardiograms and each column of data had different units.
Zhang et al. [141]	The results demonstrated an accuracy of 99.15% to diagnosis of normal rhythm, and 99.27% to diagnose atrial fibrillation. This method appeared to be sufficiently reliable for clinical use.	Some individual labels did not have enough data to adjust the parameters of the proposed method.
Abdeldayem et al. [142]	The best result was obtained by proposed time-frequency SVM model, with a maximum accuracy of 99.81%, sensitivity of 98.17%, and specificity of 99.98%	N/D
Ebrahimzadeh et al. [143]	The proposed algorithm achieved results superior to those of previously developed methods in terms of sensitivity and specificity. The authors believe the proposed can be used by doctors, if developed into an early detection system for the idiopathic onset of paroxysmal atrial fibrillation, to alert patients prior to the occurrence of the event.	The results reported by the authors lack a prospective head-to-head evaluation using clinically derived, real-world data.
Gomes et al. [145]	The results demonstrated that classification models constructed from a more relevant attributes subset, selected through an FS technique, tend to improve the quality of the models generated significantly. The proposed method could aid in the construction of classification models that assists medical specialists.	N/D
Hammad et al. [23]	The results showed that the proposed method could be used to perform real-time classification of ECG signals. This method could serve as a tool to help clinicians in confirming their diagnosis.	The proposed method was sensitive to the ECG signal quality. And, totally depended on the features values that extract from the feature extraction stage.
Hao et al. [147]	The results demonstrated that the classification accuracy of the proposed method was much higher than other methods. This method could be used to classify the high dimensional data, and, also, could be applied to the fault diagnosis.	N/D
Iqbal et al. [148]	The results obtained were satisfactory in all types of subjects, with a high accuracy. The proposed method was a significant contribution to the diagnosis of special cases of myocardial infarction.	The proposed method just covered the time domain features for patterns matching.
Liu et al. [149]	The proposed method achieved a high accuracy value, in the classification of heart disease, in a short period. When compared with other methods, long short-term memory always achieved better results	N/D
Mukherjee et al. [25]	The proposed method achieved an overall F1 Score of 83%, for the classification of normal rhythms and atrial fibrillation achieved a higher accuracy, but the detection of other types of abnormal rhythms was weak	The proposed method was not good at detecting different types of abnormal rhythms, except atrial fibrillation.
Raj et al. [151]	The proposed method, when validated using the MIT-BIH arrhythmia database, achieved higher accuracy than other existing methodologies. This method has the potential to be utilized in hospitals for on-line monitoring of continuous long-term heartbeat assessment and compressed sensing based tele-monitoring applications.	The proposed method requires more memory, the optimization technique takes a long time to tune the classifier, utilizes fixed windows for the heartbeats, and for confirmation of the method, the authors would still need to test the method in a clinical situation on patients.
Raj et al. [152]	The proposed methodology was evaluated under two analysis schemes, category-based and personalized scheme, and the performance achieved under both proves this method to be an efficient, low computational complexity and fast solution for automated classification of cardiac arrhythmias.	The proposed method uses constant windows for determining the length of ECG signals, which can vary depending on the individual, takes a long amount of time in the training and optimization stages, and is still in need of testing in a clinical situation on patients.
Warrick et al. [153]	The results showed that the proposed method yielded superior classification performance compared to a single base model.	The proposed method was not used in technical and clinical questions.
Wu et al. [154]	The result of proposed method achieved nearly 100% accuracy in the normal and ventricular ectopic beat (VEB) predictions, thus could provide flexibility to improve a wearable device's user experience and reduce its cost.	The disease classifier performance could be further strengthened with the help of increasing diversity and sizable labeled datasets.
Xu et al. [156]	The proposed method fails in improving the accuracy, sensitivity, and specificity values, but is believed by the authors to have better generalization ability. This algorithm could be used for monitoring and prevention of atrial fibrillation, which has great practical meaning.	More data could be used.
Zhang et al. [157]	The proposed model achieved a diagnosis sensitivity of 98.37%, diagnosis specificity of 99.19%, and diagnosis accuracy of 98.92%.	This work was established on three types of ECG beats only and the number of samples and type of signals need to be increased. Another limitation did not analyzing ECG beats in more leads.
Acharya et al. [158]	The results showed that the HOS features extraction method gave better results compared to other methods to identify coronary artery disease.	N/D
Andreotti et al. [159]	The feature-based classifier obtained an F1 score of 72.0% on the training setal, and 79% on the hidden test set. Similarly, the convolutional neural network scored 72.1% on the augmented database and 83% on the test set.	By ignoring noisy segments during training, was noticed a clear decrease in performance and on the final competition ranking differed significantly from the ranking during the test phase. This suggests that the split for the TEST-DB was sub-optimal and not representative of the method's performance
Couceiro et al. [160]	The results demonstrated that the extracted features were relevant to this topic and the algorithm was able to achieve better discrimination performance when compared to the previously methods.	N/D
Dolatabadi et al. [161]	This study showed that methods which were based on the feature extraction of the signals were an appropriate approach to predict the health situation of the patients. The proposed method was suitable for clinicians and could be installed in the hospitals to detect coronary artery disease automatically using HR signals.	N/D
Khatun et al. [162]	The results presented 99.7% 10-fold cross-validation accuracy with overall 99.41% sensitivity and 100% specificity from lead V4 in separating normal, different myocardial infarction and arrhythmia patients.	Due to a low number of records (440), was not applied the final model to an independent dataset.
Pławiak et al. [163]	From this study the authors were able to conclude that the best evolutionary-neural system based on the SVM classifier achieved a sensitivity of 17 myocardium dysfunctions at a level of 90.20%, an accuracy = 98.85%, and a specificity = 99.39%. Against the background of the current scientific literature, these results represent some of the best results obtained.	This method doesn't apply a completely subject-oriented validation scheme, and it does not allow for the possibility of analyzing ECG signal fragments that contain more than one class type.
Pławiak et al. [164]	The results obtained confirm that the proposed method was efficient and fast for recognition of myocardium dysfunctions.	The proposed method had a lower recognition sensitivity for heart disorders.
Plesinger et al. [165]	The resultant F1 score measured using hidden test set (3,658 recordings) of proposed based on neural networks method was 0.81 (normal 0.91, AF 0.80, OA 0.74).	The results showed demonstrated the necessity of using features based on QRS detection.
Shimpi et al. [166]	Support Vector Machine classifier model obtained the best results for classifying arrhythmia achieved accuracy of 91.2%.	N/D
Soliński et al. [168]	The final result in PhysioNet Challenge 2017 equaled 0.77 in overall F1 score. The F1 score of the signal classification as normal was 0.86, Atrial Fibrillation 0.78 and other rhythms 0.66.	N/D
Tan et al. [169]	The results, using the 8-layer stacked proposed method, achieved highest diagnostic performance. This method had the potential to be deployed in clinical settings to assist cardiologists to diagnose ECG signals.	Was not installed in a portable device, so was difficult to use for the experts.
Warrick et al. [170]	The proposed model and no explicit feature selection, 10-fold stratified cross-validation gave an overall F-measure of 0.83.10 ± 0.015 on the held-out test data (mean ± standard deviation over all folds) and 0.80 on the hidden dataset of the Challenge entry server.	This model could be refined by applying an ensemble deep learning framework to decrease information loss and overfitting problems, and to overcome the class imbalance problem.

### 4.3 Final remarks

This systematic review proved the possible impacts of ECG data in combination with machine learning when the subject is the identification of cardiovascular diseases with ECG data. In 2022, due to the world's problems, these types of diseases do not have the same importance as before. Cardiovascular diseases are among the most deadly, representing 32% of global deaths [1]. Developing a method to help people prevent cardiovascular diseases is highly recommended since the level of danger is one of the highest.

The most used databases are MIT-BIH arrhythmia and PhysioNet databases, where the most used methods are CNN and SVM. In general, the accuracy of CNN is higher than the SVM classifier. In the future, we propose adapting a technique that connects the methodology of CNN with improved accuracy and training speed for automatically learning newly labeled data. Also, combining different databases for the training and testing stages may improve the accuracy of multivariate data.

After a deep analysis of the sixty-nine studies presented in this systematic review, we can find answers to our main questions. Regarding RQ1, “Which types of sensors can be used to track different diseases?” there are some valuable sensors. In this systematic review, we saw the usage of 1-lead to 12-Lead ECG. These sensors showed much reliability when considering these types of diseases. 1-Lead ECG sensors can be used for basic monitoring and the 12-Lead ECG sensors for deeper monitoring.

Concerning RQ2, “Which ML/DP methods are mainly used primarily to support automatic analysis of ECG data?” in general, for all studies presented, the usage of deep learning methods had a significant role when the theme was the interpretation of ECG data. The convolutional neural network (CNN) is the one that made the most appearances, right followed by the deep neural network (DNN) and support vector machine (SVM). It is essential to mention that k-nearest neighbors (KNN) and Long Short-Term Memory have also been considerably used.

Regarding RQ3, “Which diseases are mostly studied with the datasets available online?”, we have seen a lot of different datasets used in these studies, counting thirty different datasets in total. Still, there is one that stands out the most. The MIT-BIH arrhythmia is the most used and related to arrhythmia disease. There is another MIT-BIH, which is directly linked to atrial fibrillation. Cardiovascular disease is studied a lot as well, appearing in several different datasets, for example, BIDMC congestive failure, 2017 Physionet/CinC, MITH-BIH normal sinus rhythm, and S.- Petersburg Institute of Cardiology Technics.

And finally, in RQ4, “What are the challenges related to the monitoring of different diseases with sensors?”, we have identified that the use of ECG sensors, which currently cost decrease, can be used for the constant monitoring of different diseases. The main challenge is related to the acceptance of the technology by the population for the measurement. However, with the use of information technologies, the privacy and security of the data are vital for the exchange of information between healthcare professionals and patients. Lastly, with the help of the ECG sensors, another challenge appears related to patient empowerment. The different models and techniques implemented in the various studies could be instrumented, thus optimizing the control and autonomy of the patient's health and treatment.

## 5 Conclusions

The 103 studies were meticulously selected in this review based on the inclusion criteria and subsequently analyzed. The review identified the sensors used to discover cardiovascular diseases, the most used ML/DP methods, the importance of the relation between the methods and sensors used, and which databases contribute the most with helpful information.

Since these diseases are one of the most dangerous, it is essential to mention that even if these sensors and methods are highly reliable, there is always room for improvement. Preventing these diseases is not always predictable, but with the help of sensors and AI-based methods such as ML and DL, we can get around the situations.

As future work, this systematic review intends to idealize a new solution for the remote identification of diseases related to ECG data and different automatic prescriptions of various medicines or treatments to reduce the problems associated with the high affluence of the healthcare institutions, giving tools to promote the independence of the population.

## Author contribution statement

Hanna Vitaliyivna Denysyuk: Analyzed and interpreted the data; Wrote the paper.

Rui João Pinto: Analyzed and interpreted the data; Wrote the paper.

Pedro Miguel Silva: Analyzed and interpreted the data; Wrote the paper.

Rui Pedro Duarte: Analyzed and interpreted the data; Wrote the paper.

Francisco Alexandre Marinho: Analyzed and interpreted the data; Wrote the paper.

Luís Pimenta: Analyzed and interpreted the data; Wrote the paper.

António Jorge Gouveia: Analyzed and interpreted the data; Wrote the paper.

Norberto Jorge Gonçalves: Analyzed and interpreted the data; Wrote the paper.

Paulo Jorge Coelho: Analyzed and interpreted the data; Wrote the paper.

Eftim Zdravevski: Analyzed and interpreted the data; Contributed reagents, materials, analysis tools or data; Wrote the paper.

Petre Lameski: Analyzed and interpreted the data; Wrote the paper.

Valderi Leithardt: Analyzed and interpreted the data; Wrote the paper.

Nuno M. Garcia: Analyzed and interpreted the data; Wrote the paper.

Ivan Miguel Pires: Analyzed and interpreted the data; Wrote the paper.

## Funding statement

This work was supported by National Funds through the Fundação para a Ciência e a Tecnologia, I.P. (Portuguese Foundation for Science and Technology) by the Project “VALORIZA—Research Center for Endogenous Resource Valorization” under Grant UIDB/05064/2020. This work is also funded by FCT/MEC through national funds and, when applicable, co-funded by the FEDER-PT2020 partnership agreement under the project UIDB/50008/2020. Hanna Vitaliyivna Denysyuk is funded by the Portuguese Foundation for Science and Technology under scholarship number 2021.06685.BD. This work was also supported by Fundação para a Ciência e a Tecnologia under Project UIDB/00308/2020.

## Data availability statement

No data was used for the research described in the article.

## Declaration of interest’s statement

The authors declare no competing interests.
